# Autophagic Inhibition of Caveolin-1 by Compound *Phyllanthus urinaria* L. Activates Ubiquitination and Proteasome Degradation of β-catenin to Suppress Metastasis of Hepatitis B-Associated Hepatocellular Carcinoma

**DOI:** 10.3389/fphar.2021.659325

**Published:** 2021-06-08

**Authors:** Danping Huang, Bowen Yang, Yaoyao Yao, Mianmian Liao, Yu Zhang, Yihao Zeng, Fengxue Zhang, Neng Wang, Guangdong Tong

**Affiliations:** ^1^ Department of Hepatology, Shenzhen Traditional Chinese Medicine Hospital, The Fourth Clinical Medical College of Guangzhou University of Chinese Medicine, Shenzhen, China; ^2^ The Research Center for Integrative Medicine, School of Basic Medical Sciences, Guangzhou University of Chinese Medicine, Guangzhou, China; ^3^ Department of Medical Biotechnology, School of Basic Medical Sciences, Guangzhou University of Chinese Medicine, Guangzhou, China

**Keywords:** metastasis, hepatitis B-associated hepatocellular carcinoma, compound *Phyllanthus urinaria* L., β-catenin, caveolin-1

## Abstract

Compound *Phyllanthus urinaria* L. (CP) is a traditional Chinese medicine (TCM) formula for cancer treatment in the clinic, particularly during progression of hepatitis B-associated hepatocellular carcinoma (HBV-associated HCC). Nevertheless, its anti-metastatic action and mechanisms are not well elucidated. In this study, CP was found to exert remarkable inhibitory effects on the proliferation, migration and invasion of HBV-associated HCC cells. The following network and biological analyses predicted that CP mainly targeted Caveolin-1 (Cav-1) to induce anti-metastatic effects, and Wnt/β-catenin pathway was one of the core mechanisms of CP action against HBV-associated HCC. Further experimental validation implied that Cav-1 overexpression promoted metastasis of HBV-associated HCC by stabilizing β-catenin, while CP administration induced autophagic degradation of Cav-1, activated the Akt/GSK3β-mediated proteasome degradation of β-catenin *via* ubiquitination activation, and subsequently attenuated the metastasis-promoting effect of Cav-1. In addition, the anti-cancer and anti-metastatic action of CP was further confirmed by *in vivo* and *ex vivo* experiments. It was found that CP inhibited the tumor growth and metastasis of HBV-associated HCC in both mice liver cancer xenograft and zebrafish xenotransplantation models. Taken together, our study not only highlights the novel function of CP formula in suppressing metastasis of HBV-associated HCC, but it also addresses the critical role of Cav-1 in mediating Akt/GSK3β/β-catenin axis to control the late-phase of cancer progression.

## Introduction

Hepatocellular carcinoma (HCC) is the fifth most diagnosed malignancy and the second leading cause of cancer-related death worldwide (El-Serag, 2012). Given that 60% of viral-related liver cancer cases are caused by Hepatitis B virus (HBV) all over the world, HBV infection is the leading cause for the progression and death of HCC (Koike, 2009). In particular, more than 350.000 thousand individuals worldwide are chronically infected with HBV and 650 thousand people die of HBV-associated cirrhosis or HCC every year (Marrero, 2006). Currently, there exist diverse anti-HCC therapeutical strategies, which mainly include early surgical treatment, radiotherapy/chemotherapy intervention, liver transplantation and targeted therapy (Hagymasi and Tulassay, 2008; Rampone et al., 2010). Despite the improvement of conventional therapies for the comprehensive treatment of HCC, the 5-year survival rate of liver cancer is still lower than 5% due to postoperative recurrence and metastasis (Li et al., 2015). A high HBV-DNA load is positively associated with HCC recurrence and HBV reactivation is an independent risk factor of 10-year survival after resection of HBV-related HCC (Hung et al., 2008; Huang et al., 2013). It has been shown that mortality in patients with HBV reactivation after curative resection of HBV-related HCC is significantly higher than that in patients without HBV reactivation (11.8% *vs*. 6.4%; *p* = 0.002) (Hung et al., 2008). Moreover, the 5-year postoperative metastasis rate is up to 45.3%, which is most likely the cause of death in patients with HCC (Zhou et al., 1994). There is a significant dose-response relationship between the incidence of HCC metastasis and HBV DNA level in patients with surgery treatment or transarterial chemoembolization (TACE) treatment (Huang et al., 2008). HBV viral load is an important risk factor for HCC metastasis (Huang et al., 2008). Therefore, the suppression of metastasis is a promising therapeutic target for HBV-associated HCC, and revealing the underlying mechanisms can boost development of strategies against HBV-associated HCC.

Aberrant activation of canonical Wnt/β-catenin pathway has always been emphasized in HBV-associated HCC, indicating advanced tumor aggressiveness and poor prognosis (Calvisi et al., 2001; Calvisi et al., 2004; Liu et al., 2016). Retrospectively, *Calvisi DF et al.* found that accumulation of Wnt ligand/receptor was a frequent issue at early hepatocarinogenesis stage of HBV-associated HCC (Calvisi et al., 2001; Calvisi et al., 2004). HBV diminished cell surface localization of *β*-catenin, resulting in the nuclear accumulation of β-catenin and activation of its target genes (von Olshausen et al., 2018). HBx-LINE1 enhanced nuclear translocation of β-catenin, subsequently leading to the induction of epithelial-mesenchymal-transition (EMT) and favoring migration and invasion tendency in HCC cell lines (Liang et al., 2016). In addition, HBV could activate PTEN/β-catenin/c-Myc signaling pathway to promote the expression of programmed cell death 1 ligand 1 (PD-L1), leading to inhibition of T cell response and eventually HBV immune evasion (Sun et al., 2020). However, there is no convincing and direct evidence clarifying the association between β-catenin signaling and metastatic process in HBV-associated HCC.

It is worth noting that traditional Chinese medicine (TCM) has gained worldwide acceptance largely due to its efficacy and safety in clinic for the prevention and treatment of cancer diseases. Moreover, its high popularity is also attributable to its multitarget, multichannel, and multisystem therapeutic characteristics. In particular, a broad range of chemicals and targets are involved in TCM formula and they exert synergistic effects in eliminating HCC cancer (Tao et al., 2015). Compound *Phyllanthus urinaria* L. (CP), including the whole plant of *Phyllanthus urinaria* L. [Phyllanthaceae], the root of *Astragalus mongholicus* Bunge [Fabaceae], the root and rhizome of *Curcuma aromatica* Salisb. [Zingiberaceae], the whole plant of *Scutellaria barbata* D. Don [Lamiaceae] and the pseudobulb of *Cremastra appendiculata* (D. Don) Makino [Orchidaceae], is such a clinical formula with favorable clinical efficacy against HBV-associated HCC. In our pilot study, we demonstrated that CP administration decreased the expression levels of upregulated gene 11 (URG11) and developmentally regulated GTP binding protein 2 (DRG2) in preneoplastic HBV-associated HCC (Tong et al., 2014). Furthermore, CP treatment inhibited the progression of HBV-associated HCC by inactivation of the HBx-SHH pathway axis (Li et al., 2019). In addition, it has frequently been reported that every single herb or most active components in CP have anti-cancer properties. For instance, *Phyllanthus urinaria L.* has been shown to suppress the HBV DNA synthesis and secretion of hepatitis B surface antigen (HBsAg) and hepatitis B core antigen (HBcAg) *via* promoting the expression of interferon beta 1 (IFN-β), cytochrome c oxidase subunit II (COX-2), and interleukin 6 (IL-6) (Jung et al., 2015). *Astragalus* polysaccharide exhibited persistent inhibitory effect on the HBV replication and the immunologic system in the transgenic mouse model integrated with HBV genome (Dang et al., 2009). Curcumin is also one of the active components of CP. A recent study has shown that curcumin possesses anti-HCC ability and shows regulatory capability toward Cav-1 and β-catenin. Curcumin and blue laser photobiomodulation could suppress the viral replication and decrease the incidence of HCC (Sun et al., 2014). Curcumin also prevented the EMT process in the diabetic rats, which might be involved in suppressing the phosphorylation of Caveolin-1 (Cav-1) at Tyr14 and increasing stabilization of Cav-1 and β-catenin (Sun et al., 2014). Furthermore, curcumin has been reported to suppress β-catenin activity in HCC cells owing to the intervention of multiprotein degradation complex and consequent interruption of the transcription of downstream genes including transcriptional regulator Myc-like (c-myc), vascular endothelial growth factor (VEGF) and cyclin D1(CCND1) (Xu et al., 2013). However, few studies have been addressed concerning the anti-metastatic effects of CP and the underlying mechanisms against HBV-associated HCC. Considering that Cav-1 has now been implicated in multiple factors including the pharmacological effects of curcumin and the regulatory mechanism of β-catenin, we further explored whether CP, a formula containing curcumin, could exert inhibitory efficacy on metastasis by regulating the function of Cav-1 and β-catenin.

In the present study, we found that CP inhibited proliferation, migration and invasion possibly *via* the Wnt/β-catenin pathway in HBV-associated HCC cells. “Formula-target-disease” network of CP was next established, revealing Cav-1 as one of the key targets of CP against HBV-associated HCC. Experimental validation further demonstrated that CP administration might promote autophagic degradation of Cav-1, therefore triggering ubiquitination and proteasome-associated degradation of β-catenin, and suppressing progression and metastasis of HBV-associated HCC *in vitro* and *in vivo*. Taken together, our study not only provided a supporting evidence for the anti-metastasis activities of CP against HBV-associated HCC, but it also addressed the novel role of Cav-1 in mediating the Akt/GSK3β/β-catenin axis during advanced cancer stages.

## Materials and Methods

### Preparation and Quality Control of Compound *Phyllanthus urinaria* L.

Herbs of CP formula include the whole plant of *Phyllanthus urinaria* L. [Phyllanthaceae] (30 g) (Barcode: 754562), the root of *Astragalus mongholicus* Bunge [Fabaceae] (15 g) (Barcode: 1261257), the root and rhizome of *Curcuma aromatica* Salisb. [Zingiberaceae] (10 g) (Barcode: 320230), the whole plant of *Scutellaria barbata* D. Don [Lamiaceae] (10 g) (Barcode: 2652051) and the pseudobulb of *Cremastra appendiculata* (D. Don) Makino [Orchidaceae] (10 g) (Barcode: 4038744). CP extract was prepared and quality control was made. An Agilent 1260 system combined with diode array detection (DAD) (Agilent, Palo Alto, CA, United States) and an Agilent C18 column (5 μm, 250 mm × 4.6 mm) with a High Performance Liquid Chromatography (HPLC) guard cartridge system (Phenomenex, SecurityGuard) were used for HPLC analysis. The mobile phases consisted of acetonitrile (A) and 0.05% (v/v) phosphoric acid (B) using a gradient program of 5–20% A in 0–15 min, 20%–40% A in 15–30 min, 40% A in 30–35 min. The flow rate was 1.0 ml/min, and column temperature was set to 30°C. The DAD detector was set at 260, 271, 350 nm. For assay of Gallic Acid, Calycosin-7-glucoside and Luteolin, the standard solutions were prepared for linearity studies. The stock solutions of Gallic Acid, Calycosin-7-glucoside and Luteolin were prepared at the suitable concentrations of methanol, respectively, then diluted with methanol to different concentrations. Ten microliter of these solutions were injected for HPLC analysis and the calibration curves were constructed by plotting the peak areas. For assay of Gallic Acid, Calycosin-7-glucoside and Luteolin of CP, CP (0.1 g) was accurately weighed in a 100 ml Erlenmeyer flask. Methanol (10 ml) was added to the Erlenmeyer flask and the mixture was sonicated for 30 min. The extract was normalized to 10 ml by adding additional methanol. Then extracting solution was filtrated through 0.2 μm membrane filter. The solution (10 μl) was injected for HPLC analysis (Supplementary Figure S1; Li et al., 2019).

### Cell Culture

Three human liver cancer cell lines (HepG2, SMMC-7721, Huh-7) and one normal hepatic cell line HL-7702 were obtained from the American Type Culture Collection (Manassas, VA, United States). HepG2-HBx, an HBV associated HCC cell line, was created in our previous study (Li et al., 2019). HepG2, HepG2-HBx, SMMC-7721 and Huh-7 were cultured in Dulbecco’s Modified Eagle Medium (DMEM) supplemented with 10% fetal bovine serum (FBS), 1% penicillin and 1% streptomycin (Gibco Life Technologies, Lofer, Austria). HL-7702 was maintained in 1640 medium supplemented with 10% FBS, 1% penicillin and 1% streptomycin (Gibco). All cell lines were incubated in a humidified incubator with settled parameters (37°C, 5% CO_2_).

### Cell Proliferation and Colony Formation Assays

Cell proliferation ability after CP treatment was detected by the Cell Counting KIT-8 (CCK-8, KeyGEN BioTECH, Nanjing, China) according to normalization protocol of the instruction. Briefly, a total of 4 × 10^3^ cells suspended were seeded in each well of 96-well plates and incubated overnight. Then, cells were dealt with different concentrations of CP extracts for 24, 48 and 72 h. The CCK-8 cell proliferation reagent (10 µl) was added to each well and cells were incubated for 4 h. Cell proliferation ability curves and the half maximal inhibitory concentration (IC50) values of CP in different cells were managed using GraphPad Prism 8.0 software and SPSS software. Colony formation assay was performed in 6-well plate. A total of 1 × 10^3^ cells suspended were seeded in each well and incubated for cell attachment. Different doses of CP extracts were added to the medium in each well. After 4 h, fresh complete medium was used to replace the cultured medium and the cells were continued to culture for 2 weeks. Subsequently, the colonies were fixed with 4% paraformaldehyde and stained with 0.5% Crystal violet for further image visualization.

### Transfection of Plasmid and Small Interfering RNA

Upregulated gene 11 (URG11), a gene upregulated by Heptatitis B Virus X protein (HBx), was identified as an oncogene promoting hepatocarcinogenesis (Lian et al., 2003; Lian et al., 2006; Du et al., 2010). Previous study has reported that HepG2 cell line stably over-expressing URG11 can be served as an HBV-associated cell line (Yuan et al., 2012). Here, we established HepG2-URG11 cell line for further molecular biology exploration of HBV-associated HCC. For transfection of plasmid, the pENTER-URG11, pcDNA 3.1(C)-Cav-1 and scrambled plasmids were obtained from Vigene Company (Jinan, China) and subsequently transfected into target cells using LipoFiter™ reagent (Hanbio Biotechnology Co, LTD. Shanghai, China). After incubation for 24 h, transfection reagent was removed. Transfected cells were cultured with fresh complete medium and screened for 2 weeks using 10 μg/ml puromycin (Invitrogen). Remaining transfected cells were further expanded for next studies. β-catenin specific siRNA as well as negative control siRNA (NC siRNA) were also obtained from Vigene Biosciences and transfected into cells using X-tremegene siRNA transfection reagent (Roche Diagnostics, Shanghai, China) according to the manufacturer's guidelines. After incubation for 24 h, transfection reagent was replaced by fresh complete medium.

### Wound Healing Assay and Transwell Assay

Migration ability was accessed by wound healing assay. We planted 4 × 10^5^ cells into a 6-well plate. When the cells permeated 90% of the plate, we scratched a wound in cell monolayer by a 1 ml pipette tip. Subsequently, cells were treated with CP with different concentrations for 48 h. Images of wounds were collected by an inverted microscope at 0, 12, 24, 48 and 72 h. Migration ability was calculated by measuring the wound confluence parameter. Invasive ability of cells was accessed by transwell assay. The transwell chambers were prepared with a layer of matrigel (no.354248, Corning, New York, United States) in advance and placed in a 24-well plate which contained complete cultured medium (10% FBS) at the bottom. A total of 8 × 10^4^ cells were seeded into upper transwell chambers. Serum-free medium (300 μl) with or without CP extract was added into the upper chambers. After 24 h, a cotton swab was used to remove the cells of the upper surface. Then the cells on the bottom surface were fixed with 4% formaldehyde solution, followed by 0.5% hematoxylin solution staining for 20 min. Images of invaded cells were captured with an inverted microscope and number of invaded cells was counted.

### Western Blotting

Protein extraction was conducted by radioimmunoprecipitation lysis buffer (RIPA) (Sigma) with protease inhibitor cocktail (Roche Diagnostics) on ice. Protein quantification was performed with the bicinchoninic-acid assay kit (Thermo Fisher Scientific, Bonn, Germany). Equal amount of protein lysates (30 µg) was loaded into each lane of sodium dodecyl sulfate polyacrylamide gel electrophoresis (SDS-PAGE), and then transferred to a polyvinylidene fluoride microporous membrane (Millipore, Billerica, MA, United States). Membranes were blocked with 5% milk at room temperature for 2 h, followed by primary antibodies incubating overnight at 4°C and secondary antibodies incubating for 1 h at room temperature. Visualization of the protein band was performed by using the enhanced chemiluminescence detection reagents (Tanon, Shanghai, China). Primary antibodies used in our study contained Cav-1(16447-1-AP, Proteintech, Chicago, United States), N-cadherin (22018-1-AP, Proteintech, Chicago, United States), E-cadherin (20874-1-AP, Proteintech, Chicago, United States), Vimentin (10366-1-AP, Proteintech, Chicago, United States), URG11 (BS2170R, Beijing, China), β-catenin (66379-1-Ig, Proteintech, Chicago, United States), *P*-Akt (AF0016, Affinity, United States), Akt (60302-2-Ig, Proteintech, Chicago, United States), *P*-GSK3β (AB11002, AbSci, Baltimore, United States), GSK3β (BF0695, Affinity, United States) and β-actin (No.3700S, Cell Signaling Technology, CST, Boston, MA, United States).

### Real-Time Polymerase Chain Reaction

Briefly, total RNA of samples was extracted using TRIzol reagent (Invitrogen, Carlsbad, CA, United States) and reverse transcription reaction was performed by PrimeScriptTMRT reagent Kit with gDNA Eraser (RR047A, TaKaRa). RT-PCR analysis was carried out using the SYBR Premix Ex Taq (Takara, Japan) according to the manufacturer’s protocol and the signal was determined by the ABI Quant Studio 7 Flex Real-Time PCR System (Applied Biosystems, Foster City, United States). Relative target mRNA expression was calculated using 2-ΔΔCt method and normalized to the internal control. Primers were synthesized by Shenggong Bioengineering Technology Limited (Shanghai, China).

### Establishment of the Herb–Ingredient–Target Interaction

Specific chemical ingredients of each herb in CP formula were searched in the Traditional Chinese Medicine Systems Pharmacology Database (TCMSP, https://tcmspw.com/tcmsp.php) and the Traditional Chinese Medicine Integrated Database (TCMID, http://www.megabionet.org/tcmid/). Oral bioavailability (OB) ≥ 30% and drug likeness (DL) ≥ 0.18 were set as criteria for screening potential ingredients. Visualization of ingredient-target network of each herb was performed by Cytoscape software (version 3.2.1).

### Gene Ontology and Pathway Enrichment Analysis

Gene expression data of HBV-associated HCC was collected from the NCBI GEO database (http://www.ncbi.nlm.nih.gov/geo). Dataset GSE44074, which contains the expression data of 17 HBV-associated HCC samples and 71 non-tumor liver specimens, was selected for further analysis. GEO2R online tool (http://www.ncbi.nlm.nih.gov/geo/geo2r/) was used to analyze the differently expressed genes (DEGs). *p* value ≤ 0.05 and fold change (FC) ≥ 1.5 were set as criteria for screening DEGs. Intersection genes between DEGs and targets of CP formula were calculated by Venn diagram (http://bioinformatics.psb.ugent.be/webtools/Venn/), then delivered into Retrieval of Interacting Genes (STRING) database for further protein-protein interaction (PPI) analysis. In addition, enrichment analyses, including gene ontology (GO) and Kyoto Encyclopedia of Genes (KEGG) pathway enrichment, were performed by DAVID database (http://david.abcc.ncifcrf.gov/). For Go annotation, three terms including biological process (BP), cellular component (CC), and molecular function (MF) were analyzed. *p* value < 0.05 was recognized as statistically significant.

### Cell Immunofluorescence

Cell immunofluorescence was used to determine the expressions of Cav-1 and β-catenin. Briefly, suspended cells were seeded on cover slips inside the 24-well plates. After drug administration, cells were fixed with 4% paraformaldehyde and permeabilized with 0.2% Triton X-100. Cell membrane was blocked by goat serum for 60 min. Following incubated with primary antibody of Cav-1 or β-catenin (Cell Signaling Technology, Beverly, MA, United States) at 4°C overnight, cells were marked by fluorescence-conjugated secondary antibodies for 1 h at room temperature in the dark. Cell nucleus was labeled with 0.1% 4′, 6-diamidino-2-phenylindole (DAPI, Sigma-Aldrich) for 15 min and fluorescence signals were detected by LMS710 confocal microscope (ZEISS).

### Coimmunoprecipitation Analysis

Protein extraction and quantification were conducted according to the protocol of western blotting. We obtained 2000 μg cell protein (750 μl) from 1 × 10^7^ cells. Protein supernatant (50 μl) was boiled with 5×Laemmli SDS-sample buffer for 5 min and served as the input sample. The remaining supernatant was incubated with the coupled antibody overnight at 4°C. Then the sample pre-incubated with antibody was moved into Capturem™ IP and Co-IP Column to wash and elute the immunoprecipitated complex according to the manufacturer's protocol (Capturem™ IP and Co-IP Kit, Takara, United States) (Cat. No. 635721). Finally, the samples were analyzed using SDS-PAGE and western blot. Antibodies of coimmunoprecipitation analysis included β-catenin (66379-1-Ig, Proteintech, Chicago, United States) and Ubiquitin (No.10201-2-AP, Proteintech, Chicago, United States).

### Animals and Experimental Design

Animal experiments in our study have been reviewed and approved by the Animal Care and Use Committee of Guangzhou University of Chinese Medicine (the Ethics Approval Number: 20210324004). To study the *in vivo* anti-cancer effect of CP on HBV-associated HCC, a total of 48 BABL/c nude mice were purchased from Medical Experiment Center of Guangdong Province (Guangzhou, China). All animals were housed under specific pathogen-free conditions (23 ± 1°C; 50 ± 10% humidity, 12 h light/dark cycle) with free access to standard food and water. To establish the tumor xenograft model, mice were subcutaneously injected in the right flank with 1 × 10^7^ tumor cells. The mice were randomly divided into four groups. Each group was fed as follow: HepG2-NC group and HepG2-HBx group were treated with normal saline. CP groups bearing with HepG2-HBx cells were administrated with a high dose (625 mg/kg) and low dose (300 mg/kg) of CP, respectively. Our previous study has performed *in vivo* experiments to validate the efficacy of CP (Li et al., 2019). Result showed when compared with the HepG2-HBx model group, the tumor inhibition rates for CP-625 mg/kg and CP-300 mg/kg were 72.64 ± 7.23% (*p* < 0.01) and 28.25 ± 10.36% (*p* < 0.001), respectively, indicating that CP exerted significant inhibitory ability for HBV-related HCC in doses of 300 mg/kg and 625 mg/kg. Meanwhile, alteration of the mice weight had no statistical significance between HepG2-HBx model group and CP treatment groups (300 mg/kg and 625 mg/kg), suggesting that CP (300 mg/kg and 625 mg/kg) had no obvious toxic side effects on nude mice. Therefore, we continued to apply the doses of 300 mg/kg and 625 mg/kg in this study to further verify the function of CP on tumor growth and metastasis *in vivo* (Li et al., 2019). Tumor volume and body weight of mice were recorded every three days. The mice were euthanized on day 34 and tumors along with other organs were collected. Experiments were performed two times in duplicates. For the *in vivo* lung metastasis assay, the lateral tail vein of the mouse was subcutaneous injected with 1 × 10^6^ tumor cells. Animal grouping was the same as described above. Four weeks later, animals were euthanized, and the lung of each mouse was removed and fixed for next histological examination.

Zebrafish xenotransplantation model was established according to the protocol provided by *Neng Wang et al.* for the evaluation of metastasis (Wang N. et al., 2019). HepG2-HBx cells were first labeled red fluorescence with 1,1′-Dioctadecyl-3,3,3′,3′-tetramethylindocarbocyanine perchlorate (DiI, Sigma-Aldrich). A total of 200 DiI-labeled HepG2-HBx cells then were injected into the perivitelline space (PVS) of 2 days post fertilization (dpf) zebrafish embryos with a microinjector. Juvenile zebrafish were subsequently maintained in 48-well plates and exposed to different concentrations of CP formula. Whether the cancer cells disperse to other tissue of zebrafish, especially to the specific tail-fin sites and form micro-metastasis was observed to evaluate the anti-metastasis effect of CP formula (Wang N. et al., 2019; Shahi Thakuri et al., 2020). Images were captured using a fluorescence microscopy (Nikon Eclipse C1, Tokyo, Japan) and analyzed with ImageJ software.

### Hematoxylin and Eosin Staining and Immunohistochemistry Analysis

Fresh tissues were fixed with 10% neutral formalin for 24 h, then embedded into paraffin and cut into sections with thickness of 4 μm. The sections were then deparaffinized with xylene twice for 10 min and rehydrated with a series of ethanol from 100 to 70%. With regard to H&E staining, cell nucleus staining was performed by 10% hematoxylin, while cytoplasm visualization was carried out by 1% eosin at room temperature. For immunohistochemistry analysis, methanol with 0.3% hydrogen peroxide was applied to inactivate the endogenous peroxide (30 min at room temperature). Then the sections were treated with sodium-citrate buffer at high pressure for antigen retrieval, with 10% goat serum for serum blocking. The sections were subsequently incubated with primary antibodies against β-catenin, Cav-1, Vimentin and E-cadherin. The 3,3-diaminobenzidine (DAB) solution was applied as chromogenic agent for nuclear staining to identify the expression of protein.

### Statistical Analysis

All parameters in this research were presented as mean ± standard deviation (SD). A triplicate independent experiment was performed. SPSS 19.0 software (Abbott Laboratories, Chicago, United States) was utilized for statistical analyses. Diagrams were plotted using GraphPad Prism 8.0 software. The two-tailed Student’s t-test and one-way analysis of variance (ANOVA) were used for statistical significance of data. *p* value < 0.05 was defined as statistically significance.

## Results

### Compound *Phyllanthus urinaria* L. Suppressed Proliferation, Migration and Invasion of Hepatitis B Virus-Associated Phenotypes of Hepatocellular Carcinoma Cells

**FIGURE 1:**
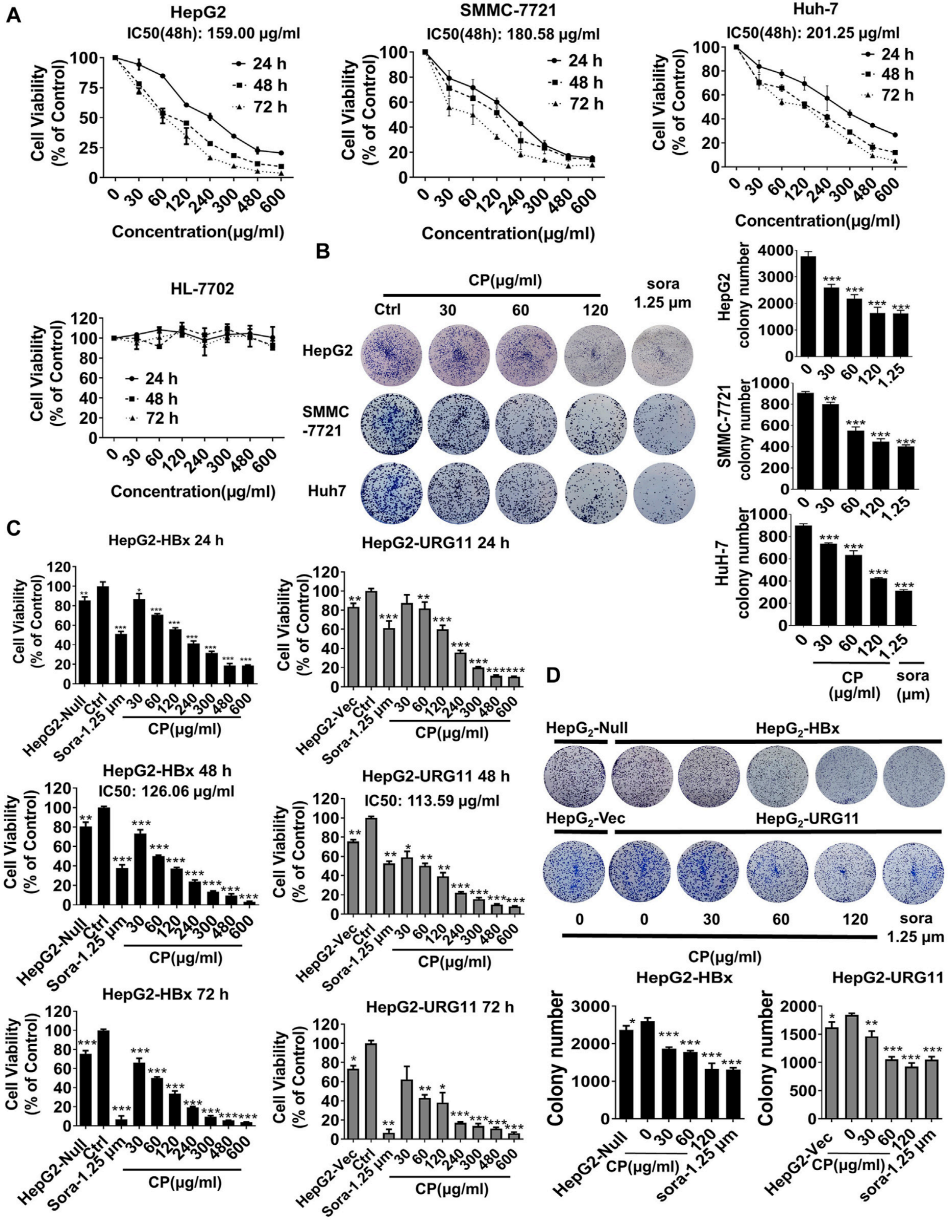
CP suppressed the proliferation of HBV-associated phenotypes of HCC cells. **(A)** CP exhibited obvious inhibition on HepG2, SMMC-7721 and HuH-7 in a time-concentration dependent manner and had no obvious effect on normal hepatic cell HL-7702. **(B)** CP significantly reduced colony size and number of HCC cells including HepG2, SMMC-7721 and HuH-7. **(C)** CP administration suppressed the proliferation of HBV-associated HCC cell including HepG2-HBx and HepG2-URG11 cells in a dose-dependent manner. **(D)** CP significantly inhibited the clone formation capability of both HepG2-HBx and HepG2-URG11 cells. All values represented the means ± SD (*n* = 3, *p < 0.05, **p < 0.01, ***p < 0.001). sora: sorafenib.

To assess the anti-cancer effects of CP on HCC cells, we first employed CCK-8 assay on several representative HCC cell lines (HepG2, SMMC-7721 and Huh-7) and a normal hepatic cell line (HL-7702). The results demonstrated that CP exhibited obvious inhibitory activities on HepG2, SMMC-7721 and Huh-7 cells in a dose-and time-dependent manner. In contrast, it exerted obscure effect on survival of normal hepatic cells HL-7702 (Figure 1A). As shown in Supplementary Table S1, CP showed the strongest inhibitory effect on HepG2 cells indicated by the highest selectivity index (SI = 9.41). This finding implied that CP showed higher cytotoxicity selectivity to HepG2 cells than to the other two cell lines. Hence, we chose the HepG2 cell line as a representative cell line for all subsequent experiments. The colony formation array was then conducted to assess long-term inhibitory effects of CP on the indicated cancer cells. It was found that CP significantly reduced colony size and number of HCC cells. The representative images of colony formation assay are shown in Figure 1B. Next, we continued to investigate the inhibitory effects of CP on HBV-associated phenotypes of HCC cells. HepG2-HBx and HepG2-URG11 cell lines were established and validated as representative HBV-associated HCC cell lines for the subsequent experiments according to the previous study (Yuan et al., 2012). For the establishment of HepG2-HBx cells, HepG2 cells were infected with lentiviral particles expressing HBx and then screened with 1.6 μg/ml puromycin for 7 days (Li et al., 2019). For the construction of HepG2-URG11 cells, the pENTER-URG11 plasmid was transfected into HepG2 cells using LipoFiter™ reagent, and stable cell line was screened by geneticin for additional 2 weeks. Compared with the parental cells, HBV-associated HCC cells exhibited a higher degree of malignant potential, particularly increased migratory and invasive abilities. Wound closure was significantly accelerated in HepG2-HBx and HepG2-URG11 cells (Supplementary Figure S2A). In addition, the expression levels of Vimentin and N-cadherin increased, while the expression level of E-cadherin decreased in both HepG2-HBx and HepG2-URG11 cells, further indicating that HBV-associated HCC exhibited stronger metastasis ability (Supplementary Figure S2B). We then investigated the effects of CP on cell proliferation of HBV-HCC cell lines. Since sorafenib was reported to protect against the metastasis of HBV-related HCC through inhibiting Raf kinase and vascular endothelial growth factor (VEGF) receptor and served as a systemic therapy for advanced HCC (Lin et al., 2016; Harding et al., 2018), the sorafenib group was included as the positive control in this study. As shown in Figure 1C, CCK-8 array revealed that either HBx or URG11 overexpression increased cell viability of HCC, while CP administration dose-dependently suppressed the growth of both HepG2-HBx and HepG2-URG11 cells. Particularly, the IC50 was 126.06 μg/ml for HepG2-HBx cells, and 113.59 μg/ml for HepG2-URG11 cells after 48 h of CP treatment. This finding was consistent with the colony formation assay, given a decreased number of CP-treated colonies in both HepG2-HBx and HepG2-URG11 cells (Figure 1D). Overall, CP limited the growth of HBV-associated HCC cells, without exhibiting considerable cytotoxicity on normal cells.

**FIGURE 2:**
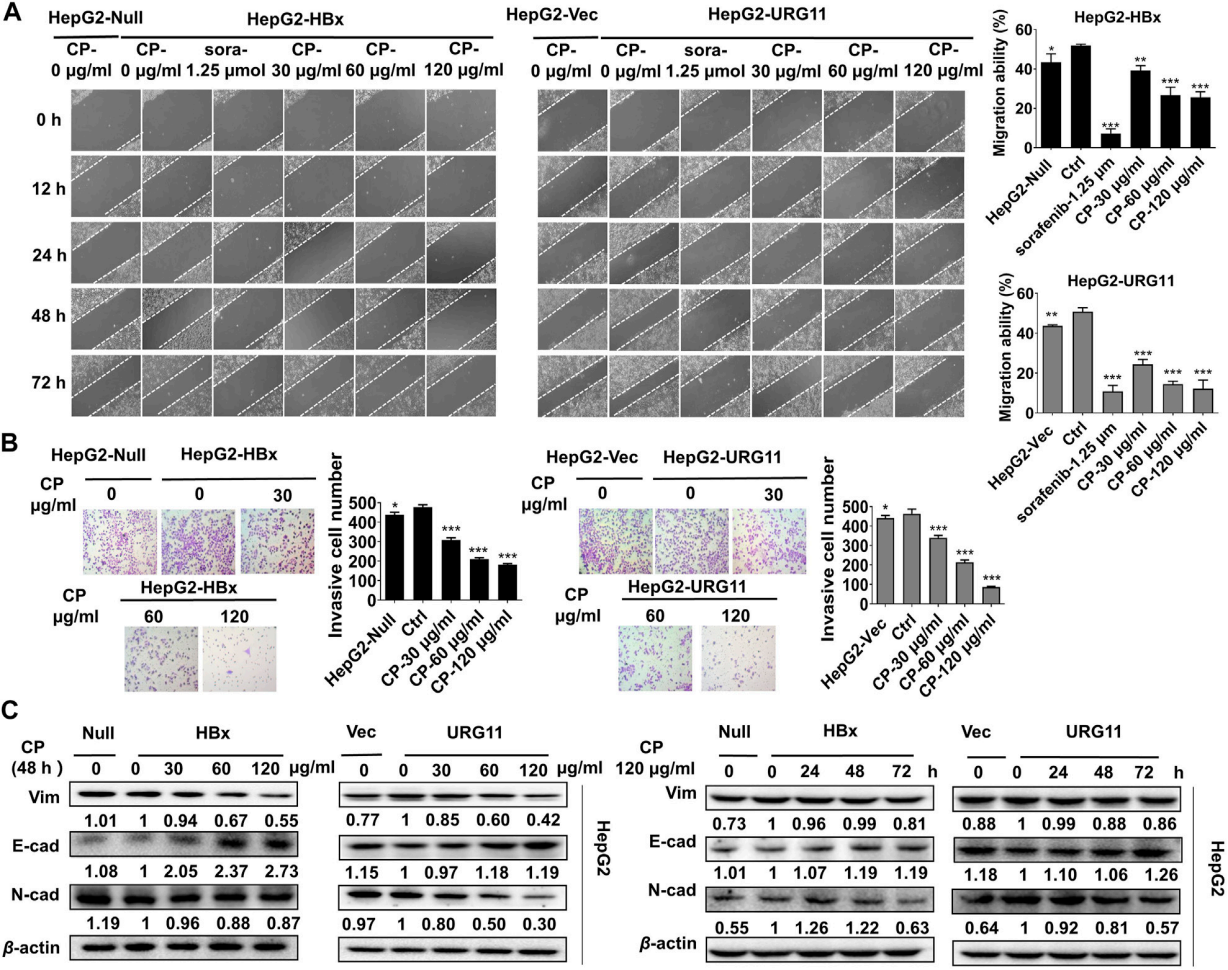
CP inhibited migration and invasion of HBV-associated HCC. **(A)** HBV-associated cells including HepG2-HBx and HepG2-URG11 following CP administration and sorafenib treatment exhibited a significant delay in wound healing compared with that of control group. **(B)** CP treatment remarkably decreased the number of HBV-associated cells that migrated across matrigel and transwell chamber. **(C)** Western blot analysis showed that CP dramatically decreased expression levels of Vimentin and N-cadherin and increased E-cadherin level in both HepG2-HBx and HepG2-URG11 cells in a time-concentration dependent manner. All values represented the means ± SD (*n* = 3, *p < 0.05, **p < 0.01, ***p < 0.001).

Next, we investigated whether CP exerted anti-metastatic effects on HBV-associated HCC. An *in vitro* scratch-wound healing assay was conducted to investigate the migration potential of HBV-associated HCC in the presence or absence of CP. Following CP administration and sorafenib treatment, the HBV-associated cells exhibited a significant delay in wound healing compared with that of the control group (Figure 2A). Transwell assay was also employed to quantify the invasive capacities of the metastatic cancer cells. We found that 24 h of CP treatment remarkably decreased the number of HepG2-HBx and HepG2-URG11 cells that migrated across the transwell chamber (Figure 2B). EMT is a phenotypic conversion process associated with cancer progression and metastasis in which epithelial cells acquire the motile and invasive characteristics and transform into mesenchymal cells (Kim et al., 2019). Western blot analysis was performed to examine the EMT-related proteins, including Vimentin, N-cadherin and E-cadherin. CP administration reduced the expression levels Vimentin and N-cadherin expressions and increased E-cadherin level at a dose- and time-dependent manner (Figure 2C). Taken together, these data indicated that CP could inhibit migration and invasion ability of HBV-associated HCC.

### Compound *Phyllanthus urinaria* L. Promoted the Destabilization of β-catenin in Hepatitis B Virus-Associated Hepatocellular Carcinoma

**FIGURE 3:**
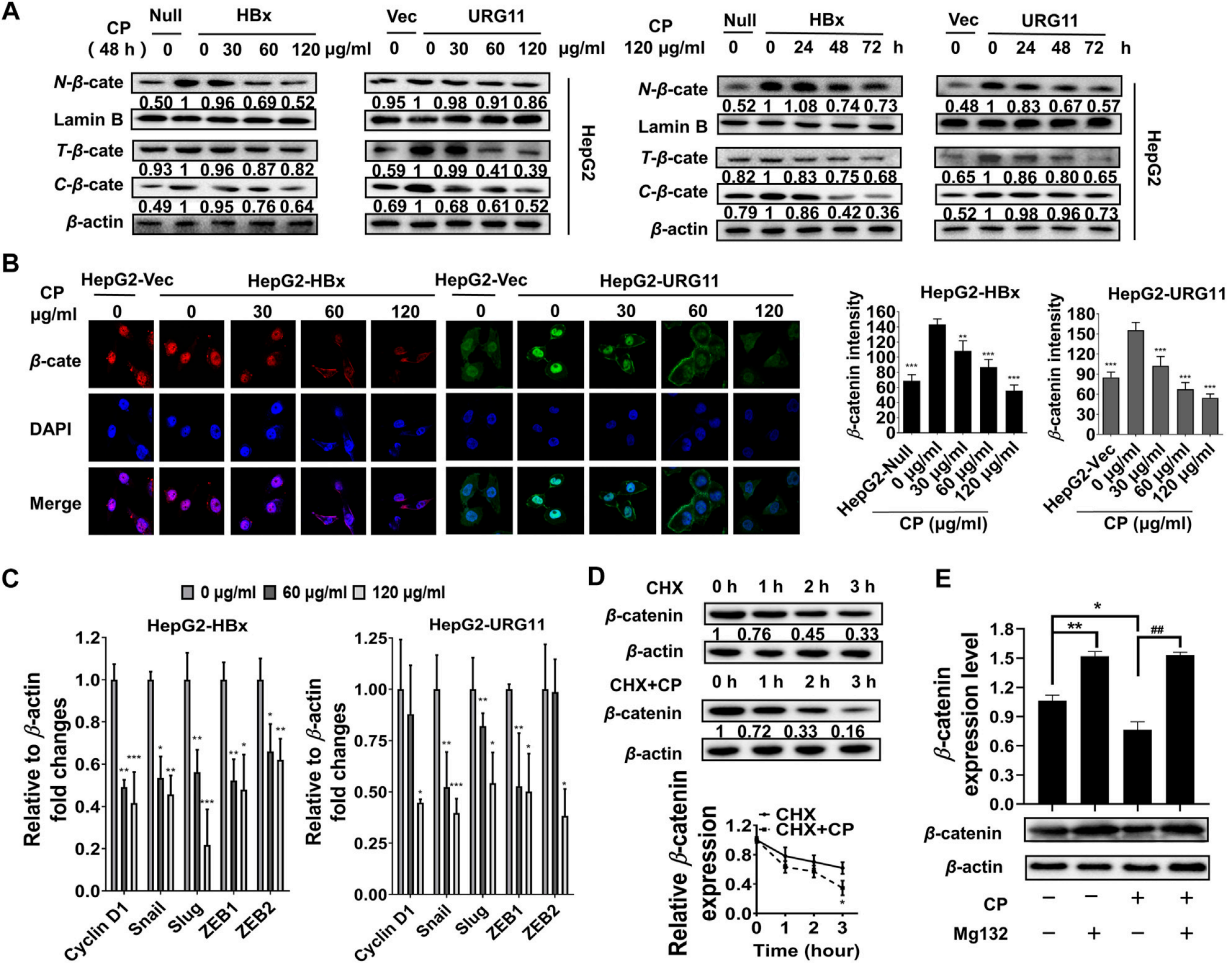
CP suppressed metastasis of HBV-associated HCC *via* promoting the destabilization of β-catenin. **(A)** CP decreased both total and fractional expression levels of β-catenin in HepG2-HBx and HepG2-URG11 cells. **(B)** Immunofluorescence analysis presented that CP inhibited the nuclear accumulation of β-catenin. **(C)** QPCR array showed that the proliferation-related TF *Cyclin D1* and the EMT-related TFs *Snail*, *Slug*, *ZEB1* and *ZEB2* were simultaneously suppressed by CP. **(D)** β-catenin downregulation was triggered in the CHX group while its degradation was remarkably accelerated in the CHX + CP group. **(E)** Meanwhile, proteasome inhibitor MG132 inhibited CP induction of β-catenin degradation. All values represented the means ± SD (*n* = 3, *p < 0.05, **p < 0.01, ***p < 0.001, ^##^ *p* < 0.01).

Previous studies showed that aberrant expression of β-catenin was a critical step associated with invasion and metastasis in HBV-associated HCC (Yuan et al., 2012; Hong et al., 2017; Mao et al., 2019). After validating the anti-metastasis effects of CP, we further investigated whether this process would be β-catenin-dependent in HBV-associated HCC. We first examined the effect of CP on the expression and subcellular localization of β-catenin. As shown in Figure 3A, both total and fractional expression levels of β-catenin were downregulated after exposure to CP in both HepG2-HBx and HepG2-URG11 cell lines. Particularly, both nuclear and cytosolic β-catenin expression were simultaneously suppressed by CP. Immunofluorescence analysis further supported our findings, showing a decrease in nuclear accumulation of β-catenin after CP treatment in both HepG2-HBx and HepG2-URG11 cells (Figure 3B). We further examined the β-catenin mRNA level in HepG2-HBx with CP treatment. As shown in Supplementary Figure S3A, we found that CP treatment did not alter β-catenin mRNA level. Since the suppressing activities of CP on nuclear expression of β-catenin might abrogate transcription of several downstream genes for β-catenin, we next detected the expression levels of the proliferation-related transcription factor (TF) *Cyclin D1* and the EMT-related TFs *Snail*, *Slug*, *ZEB1* and *ZEB2*. As shown in Figure 3C, qPCR analysis demonstrated that CP obviously downregulated the expressions of *Cyclin D1*, *Snail*, *Slug*, *ZEB1* and *ZEB2*. Previous studies reported that the activation of proteasome degradation is the primary cause for the translational regulation of β-catenin expression (Wang et al., 2014). We then examined whether suppression of β-catenin by CP was attributable to the proteasome degradation pathway. Herein, MG132 was used to inhibit proteasome function and cycloheximide (CHX) was used to inhibit the protein synthesis. As shown in Figure 3D, HepG2-HBx cells treated with CP exhibited an increased β-catenin degradation rate compared with the control group in the presence of CHX, indicating that CP accelerated β-catenin protein degradation. Since the ubiquitin-proteasome pathway is the most ubiquitous way for β-catenin protein degradation, we treated the cells with the ubiquitin–proteasome pathway inhibitor MG132. We found that MG132 inhibited CP induction of β-catenin degradation (Figure 3E), indicating that CP mediated β*-*catenin degradation *via* the proteasome-dependent pathway. Taken together, the data demonstrated that CP could activate the proteasome degradation of β-catenin.

Furthermore, it has been reported that the Akt/GSK3β/β-catenin pathway is a critical functional signaling pathway involved in the regulation of EMT and cell invasion (Wang et al., 2016). GSK-3β can be inactivated by phosphorylation at the N-terminal serine 9 (Ser9) residue, which negatively regulates the activity of the GSK-3β and therefore stabilizes β-catenin (Cole et al., 2004; MacDonald et al., 2009; Park et al., 2013; Hu et al., 2016; Xu et al., 2017; Zheng et al., 2020). The stimulation of the Wnt canonical pathway by specific ligands, inactivates GSK-3β by specific phosphorylation at Ser9, stabilizes β*-*catenin, then promotes its nucleus translocation and interaction with transcription factors of the downstream Tcf/Lef family (MacDonald et al., 2009). In this study, CP downregulated the phosphorylation of GSK3β (ser9) in a dose- and time-dependent manner, indicating that CP was an adverse factor for the stabilization of β-catenin, which might result in the proteasome degradation of β-catenin protein (Supplementary Figure S3B). Furthermore, activated Akt (*P*-Akt) was reported to be the upstream signal to phosphorylate the GSK3β at Ser9 site, resulting in the inactivation of GSK3β and stabilization of β-catenin protein (Livak and Schmittgen, 2001; Manning and Cantley, 2007; Zhang et al., 2011; Ding et al., 2016; Wang and Zhao, 2016). Herein, we found that *P*-Akt was also significantly suppressed by CP in HepG2-HBx and HepG2-URG11 cell lines (Supplementary Figure S3B), indicating that AKT inhibition might accelerate CP-mediated β-catenin degradation. To thoroughly investigate whether Akt/GSK3β signaling is responsible for the β-catenin degradation in the CP treatment group, LY294002 (LY, the AKT inhibitor) and lithium chloride (LiCl, the GSK-3β inhibitor) were used to suppress the activity of AKT and GSK-3β, respectively. LY treatment decreased the expression of β-catenin whereas LiCl treatment effectively enhanced the expression of β-catenin in the HepG2-HBx cell line in the presence of CP (Supplementary Figure S3C), suggesting that CP might promote β-catenin degradation through Akt/GSK3β signaling.

### Establishment of Network of Compound *Phyllanthus urinaria* L. Ingredient-Compound *Phyllanthus urinaria* L. Target-Hepatitis B Virus-Associated Hepatocellular Carcinoma

**FIGURE 4:**
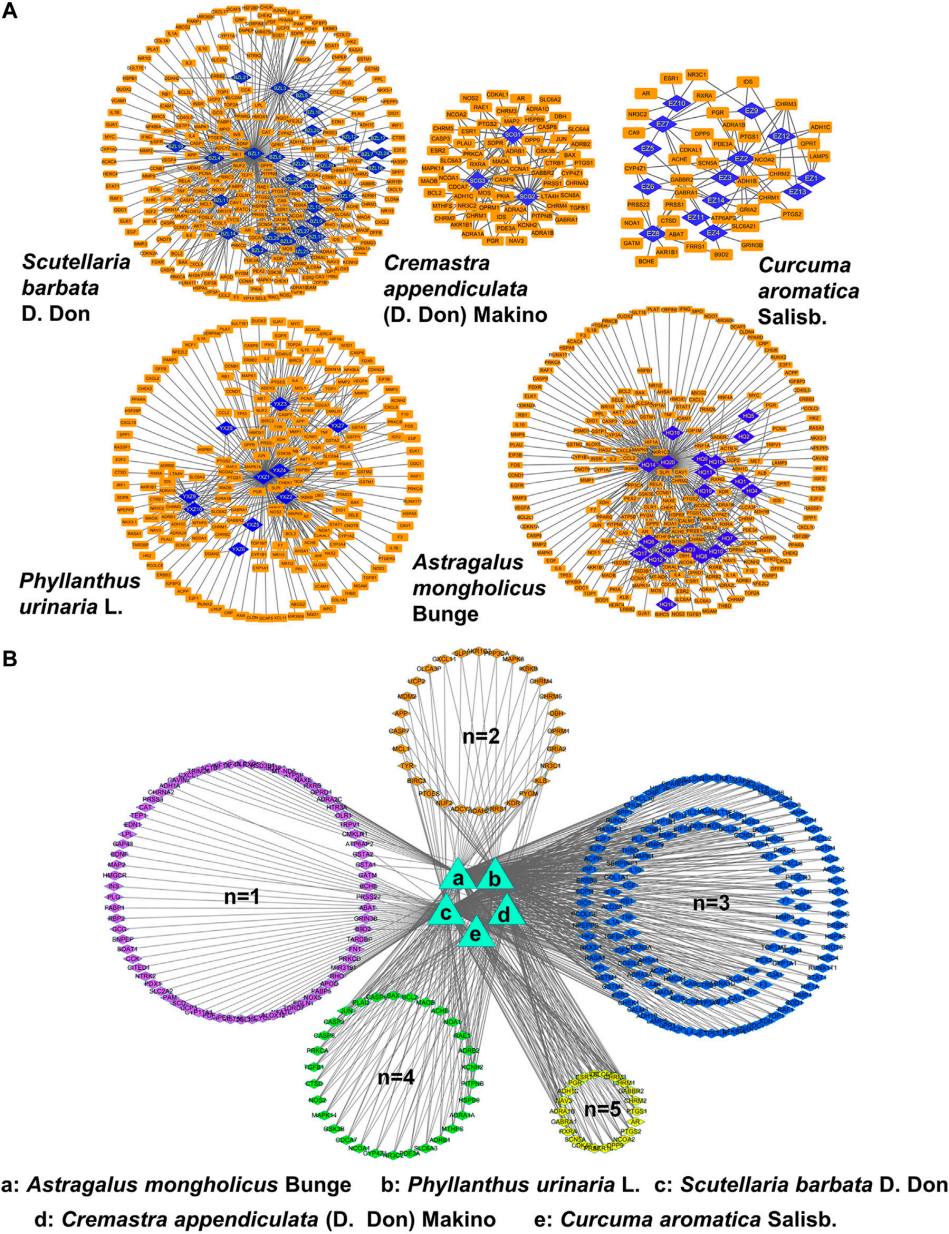
Network pharmacology analysis of CP. **(A)** Ingredient-target networks of the five herbs of CP. There are 29 major ingredients in *Scutellaria barbata* D. Don, three in *Cremastra appendiculata* (D. Don) Makino, 14 in *Curcuma aromatica* Salisb., 10 in *Phyllanthus urinaria* L. and 20 in *Astragalus mongholicus* Bunge. **(B)** Common targets networks of CP. 76 were common targets of one herb, 28 were common targets of two herbs, 142 were common targets of three herbs, 30 were common targets of four herbs and 22 were common targets of five herbs.

To unravel the anti-metastasis mechanism of CP, we performed network pharmacology analysis of CP with the criteria of OB ≥ 30% and DL ≥ 0.18. As shown in Figure 4A, ingredient-target networks were constructed for the five herbs of CP. The results revealed that 29 major ingredients in *Scutellaria barbata* D. Don, three in *Cremastra appendiculata* (D. Don) Makino, 14 in *Curcuma aromatica* Salisb., 10 in *Phyllanthus urinaria* L*.* and 20 in *Astragalus mongholicus* Bunge may play important roles in the efficacy of CP. After excluding duplicate targets, there were 296 remaining predicted target genes for the CP formula (Figure 4B). Among these candidate targets, 76 were common targets of one herb, 28 were common targets of two herbs, 142 were common targets of three herbs, 30 were common targets of four herbs and 22 were common targets of all five herbs.

**FIGURE 5:**
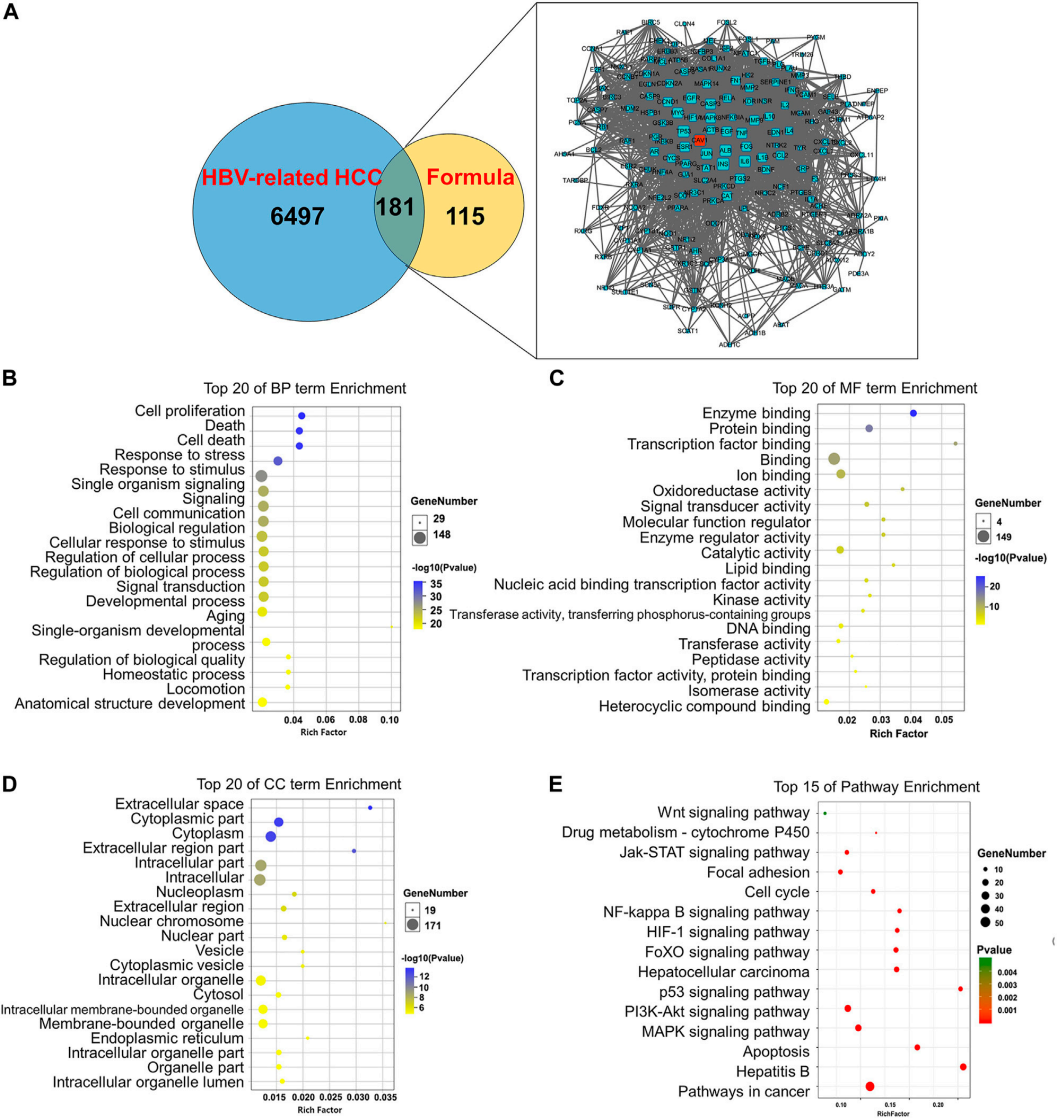
Establishment of ingredient-target-disease network of CP in HBV-associated HCC. **(A)** The Venn diagram showed a total of 181 putative CP targets for HBV-associated HCC were extracted. CAV-1 was one of the largest nodes based on the “Degree > 100”. **(B)** Pathway enrichment analysis of the 181 hub genes. **(B)** The top 20 terms of BP enrichment **(C)** The top 20 terms of MF enrichment. **(D)** The top 20 terms of CC enrichment **(E)** Pathway enrichment analysis of the DEGs.

Furthermore, we retrieved the corresponding DEGs of HBV-associated HCC from GSE44074 microarray of 17 HBV-associated HCC patients and 71 individuals with non-tumor liver specimens to reveal how the active ingredients of CP act on the HBV-associated HCC. A total of 6,497 disease-related targets were obtained after GEO2R analysis (*p* ≤ 0.05, FC ≥ 1.5). After matching the candidate target genes of CP ingredients with the disease-related targets using Venn diagram, a total of 181 putative CP targets for HBV-associated HCC were extracted. By uploading 181 candidate targets to the STRING database and setting the *p*-value to less than 1 × 10^–16^, a protein-protein interaction (PPI) was obtained. It was found that CAV-1 was one of the largest nodes based on the “Degree > 100”, indicating that CAV-1 might play a crucial role in the anti-cancer efficacy of CP (Figure 5A). We also performed GO enrichment and KEGG analysis using DAVID database to further investigate the anti-cancer mechanisms of CP in HBV-associated HCC. For GO-term analysis, the y-axis represented the corresponding GO term and the x-axis indicated the number of enriched genes. For each GO term, the first top 20 terms were presented as heatmap according to the *p*-value. The major terms of BP enrichment comprised cell proliferation, cell death, response to stress, regulation of cellular process, regulation of biological process (Figure 5B). MF analysis was mainly enriched in enzyme binding, protein binding, transcription factor binding, ion binding and oxidoreductase activity (Figure 5C). CC terms revealed that CP action was associated with extracellular space, cytoplasmic part, intracellular part, nucleoplasm and nuclear chromosome (Figure 5D). For KEGG analysis, the 181 candidate genes were interacted with multiple cancer-related pathways, among them, Wnt signaling pathway was addressed as one of top 15 enriched pathways. Besides, the anti-metastasis function of CP might be also attributable to other interfering signaling pathways, such as the MAPK signaling pathway, PI3K-Akt signaling pathway, p53 signaling pathway and so on (Figure 5E).

### Compound *Phyllanthus urinaria* L. Promoted Autophagic Degradation of Caveolin-1

**FIGURE 6:**
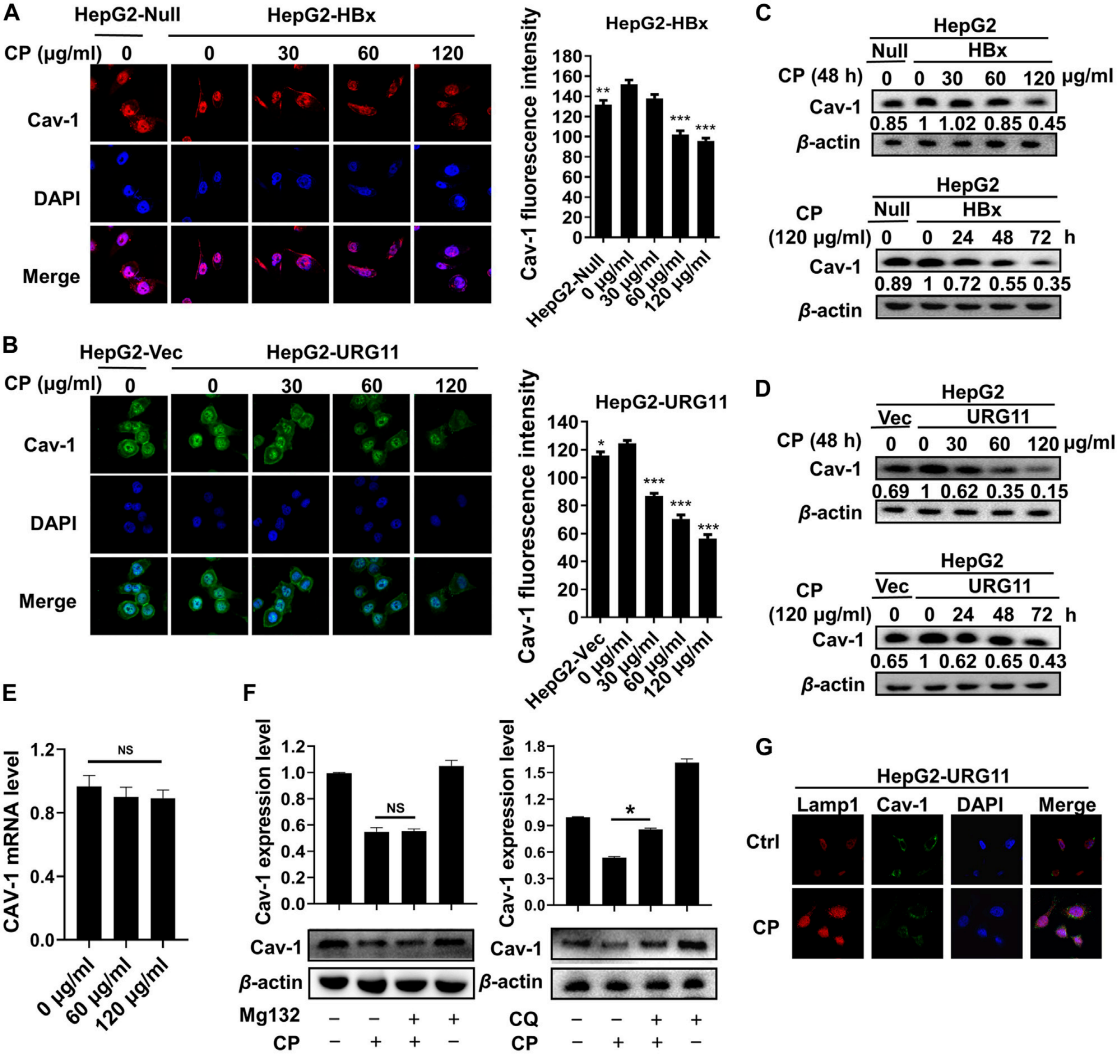
CP promoted autophagic degradation of Cav-1. **(A, B)** Immunofluorescence staining revealed that the immunosignal of Cav-1 was inhibited by CP in a concentration-dependent manner in HepG2-HBx and HepG2-URG11 cells. **(C, D)** Western blot analysis showed that CP treatment decreased the expression of Cav-1 in a dose- and time-dependent manner in HepG2-HBx and HepG2-URG11 cells. **(E)** CP have no significant effect on modulating the mRNA expression of Cav-1. **(F)** The autophagic degradation inhibitor CQ, rather than MG132, blocked the degradation process of Cav-1 by CP. **(G)** Immunofluorescence array showed that co-localization efficiency of Lamp1 and Cav-1 was increased in CP treatment group. All values represented the means ± SD (*n* = 3, *p < 0.05, **p < 0.01, ***p < 0.001, NS: *p* > 0.05 not significant).

The network pharmacology and bioinformatics analysis revealed that CAV-1 was one of the core targets of CP to antagonize HBV-associated HCC in terms of network pharmacology and bioinformatics analysis. We next evaluated the influence of CP on Cav-1 expression in both HepG2-HBx and HepG2-URG11 cell lines after the indicated CP treatment. As demonstrated in Figures 6A,B, immunofluorescence staining revealed that CP decreased Cav-1 immunosignals in both cell membrane and cytoplasm of cells. This result was further confirmed by Western blot analysis in HepG2-HBx and HepG2-URG11 cells. It was found that CP administration was associated with lower Cav-1 expression in a dose- and time-dependent manner (Figures 6C,D). To explore the molecular mechanism by which CP downregulated Cav-1 expression, we further examined Cav-1 mRNA level in HepG2-HBx with CP treatment. We found that CP treatment did not significantly regulate CAV-1 mRNA level (Figure 6E). Thus, the decreased Cav-1 protein expression might be attributable to either proteasome degradation process or autophagic degradation pathway. To distinguish whether the proteasome degradation process or autophagic degradation pathway was important for the CP effect, we firstly administrated CP with proteasome inhibitor MG132. As shown in Figure 6F (*left panel*), the statistical results showed there was no significance between CP alone group and the MG132 + CP group. MG132 treatment couldn’t restore the expression of Cav-1 in the presence of CP, indicating that Cav-1 could not be regulated by proteasome pathway. Then CP was administered either alone or in combination with the lysosomal inhibitor CQ. As shown in Figure 6F *right panel*, CQ could significantly block the decline of Cav-1 protein induced by CP, indicating that Cav-1 might be degraded *via* autophagy pathway. To further investigate the relationship between Cav-1 with autophagy in the presence of CP, we co-stained Cav-1 with the lysosome marker Lamp1 to further investigate the association between Cav-1 with autophagic degradation in the presence of CP. The protein level of Lamp1 is regarded as an indicator of autophagy (Eskelinen, 2006), which is closely correlated with autophagosomes degradation. In the last step of autophagy, under the mediation of lysosomal-associated membrane proteins Lamp1, the autolysosome contents are degraded by a series of lysosomal proteases (Wang X. et al., 2019). As expected, CP administration significantly increased Lamp1 intensity and decreased Cav-1 expression, while the co-expression of Lamp1 and Cav-1 were increased in the CP-treated group. These findings probably suggested that the CP administration led to a translocation and accumulation of Cav-1 in lysosome, and Cav-1 downregulation by CP likely occurred through autophagic degradation (Figure 6G). Overall, the data revealed that CP might elicit Cav-1 downregulation *via* the autophagy/lysosome pathway.

### Caveolin-1 Inhibition by Compound *Phyllanthus urinaria* L. Activated the Akt/GSK3β-Mediated Proteasome Degradation of β-catenin *via* Ubiquitination Activation

**FIGURE 7:**
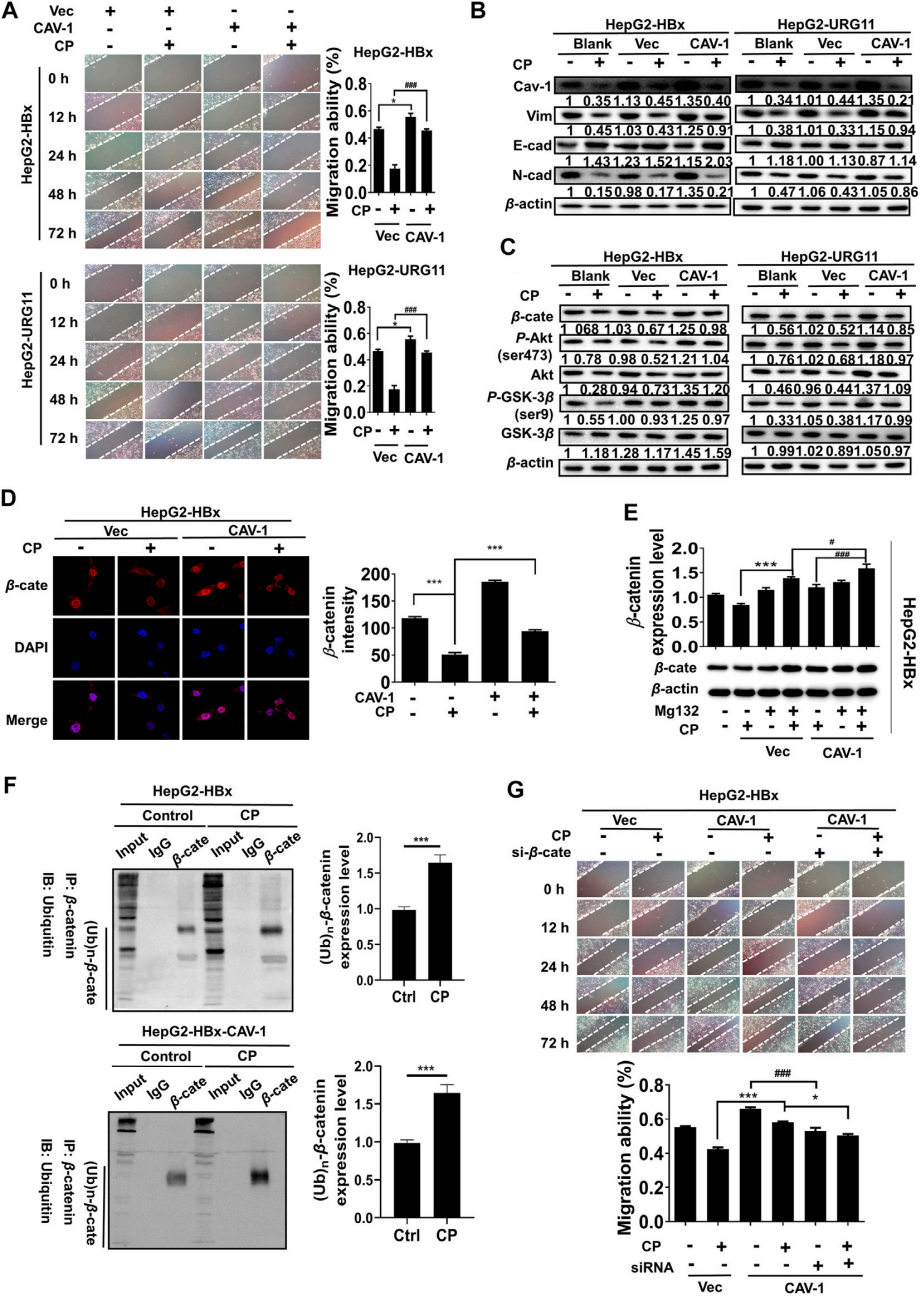
Cav-1 inhibition by CP activated the Akt/GSK3β-mediated proteasome degradation of β-catenin *via* ubiquitination activation. **(A)** Wound healing assay showed the gap widths and areas were decreased in the Cav-1-overexpression groups, while CP treatment attenuated the migration ability of HBV-associated cells. **(B)** Compared to vector control, CP attenuated the promotion effect of Cav-1 on EMT process of HBV-associated HCC. **(C)** Cav-1 increased β-catenin expression and activated Akt/GSK3β pathway. CP treatment attenuated the effect of Cav-1 on β-catenin expression and Akt/GSK3β pathway of HBV-associated HCC. **(D)** Immunofluorescence assay of β-catenin expression and distribution in the cases of high Cav-1 expression and CP treatment. **(E)** Compared with the vector group, accumulation of β-catenin was accelerated in Cav-1-overexpressed group in the presence of CP following MG132 treatment. **(F)** CP significantly enhanced ubiquitination of β-catenin in HepG2-HBx cell lines with or without Cav-1 overexpression. **(G)** Wound healing array showed that β-catenin knockdown partly abrogated the capacity of Cav-1 to promote migration in HepG2-HBx cell. All values represented the means ± SD (*n* = 3, *p < 0.05, **p < 0.01, ***p < 0.001, ^#^ *p* < 0.05, ^##^ *p* < 0.01, ^###^ *p* < 0.001).

To provide further evidence supporting the critical role of Cav-1 for the anti-metastatic activities of CP, we elevated Cav-1 expression with pcDNA 3.1(+)-Cav-1 plasmid in the HepG2-HBx and HepG2-URG11 cells for the following studies. Compared with vector groups, the migration potential was aggravated in the Cav-1-overexpression groups, while CP treatment attenuated such metastatic capabilities by increasing the gap widths and areas of both HepG2-HBx and HepG2-URG11 cells (Figure 7A). Furthermore, Cav-1 overexpression also led to increased Vimentin and N-cadherin expression as well as decreased E-cadherin level in HBV-associated HCC in the presence of CP (Figure 7B). Since we demonstrated that β-catenin downregulation by CP was at least partly responsible for its anti-metastatic potential, we next investigated the association between Cav-1 and β-catenin by observing the effects of Cav-1 upregulation on the Akt/GSK3β/β-catenin axis in the presence of CP. Western blot analysis revealed that exogenous overexpression of Cav-1 blocked the CP-induced degradation of β-catenin expression likely by increasing the phosphorylation of AKT and GSK-3β, implying a positive regulatory relationship between Cav-1 and Akt/GSK3β/β-catenin signaling (Figure 7C). This finding was confirmed by immunofluorescence assay of β-catenin expression, presenting as overexpression of Cav-1 increased the expression of both nuclear and cytoplasmic β-catenin under CP treatment (Figure 7D). We further explored whether Cav-1 overexpression was responsible for maintaining the stabilization of β-catenin by suppressing its proteasome degradation in the presence of CP. We found that the CP-induced β-catenin degradation was greatly blocked following MG132 treatment in the Cav-1-overexpressing group compared with the vector group, which resulted in the accumulation of β-catenin (Figure 7E). Since ubiquitination activation played the key role in β-catenin degradation (Liu et al., 2008), we next performed Co-IP combined with ubiquitination array in HepG2-HBx and Cav-1-overexpressing HepG2-HBx cells with or without CP. Decreased ubiquitination of β-catenin was observed in HepG2-HBx-Cav-1 compared with HepG2-HBx group, further verifying that ubiquitination pathway of β-catenin was suppressed by Cav-1 (Supplementary Figure S4A). As shown in Figure 7F, CP significantly enhanced ubiquitination of β-catenin in both HepG2-HBx and HepG2-HBx-Cav-1 cell lines. Moreover, the AKT inhibitor LY enhanced β-catenin degradation in HepG2-HBx-Cav-1 cell line after CP treatment, while the GSK-3β inhibitor LiCI attenuated the inhibitory effect of CP on β-catenin expression in HepG2-HBx-Cav-1 cells (Supplementary Figure S4B). To thoroughly investigate whether β-catenin is required for the metastasis-promoting effect of Cav-1 in HepG2-HBx cells, β-catenin was silenced in Cav-1-overexpressing HepG2-HBx cells. As shown in Figure 7G, wound healing potential was potently suppressed following β-catenin knockdown in High-Cav-1 group. Taken together, Cav-1 overexpression led to a blockage of β-catenin degradation induced by CP, subsequently exerting a metastasis-promoting role in HBV-associated HCC.

### Compound *Phyllanthus urinaria* L. Inhibited Proliferation and Migration of Hepatitis B Virus-Associated Hepatocellular Carcinoma *In Vivo* and *Ex Vivo*

**FIGURE 8:**
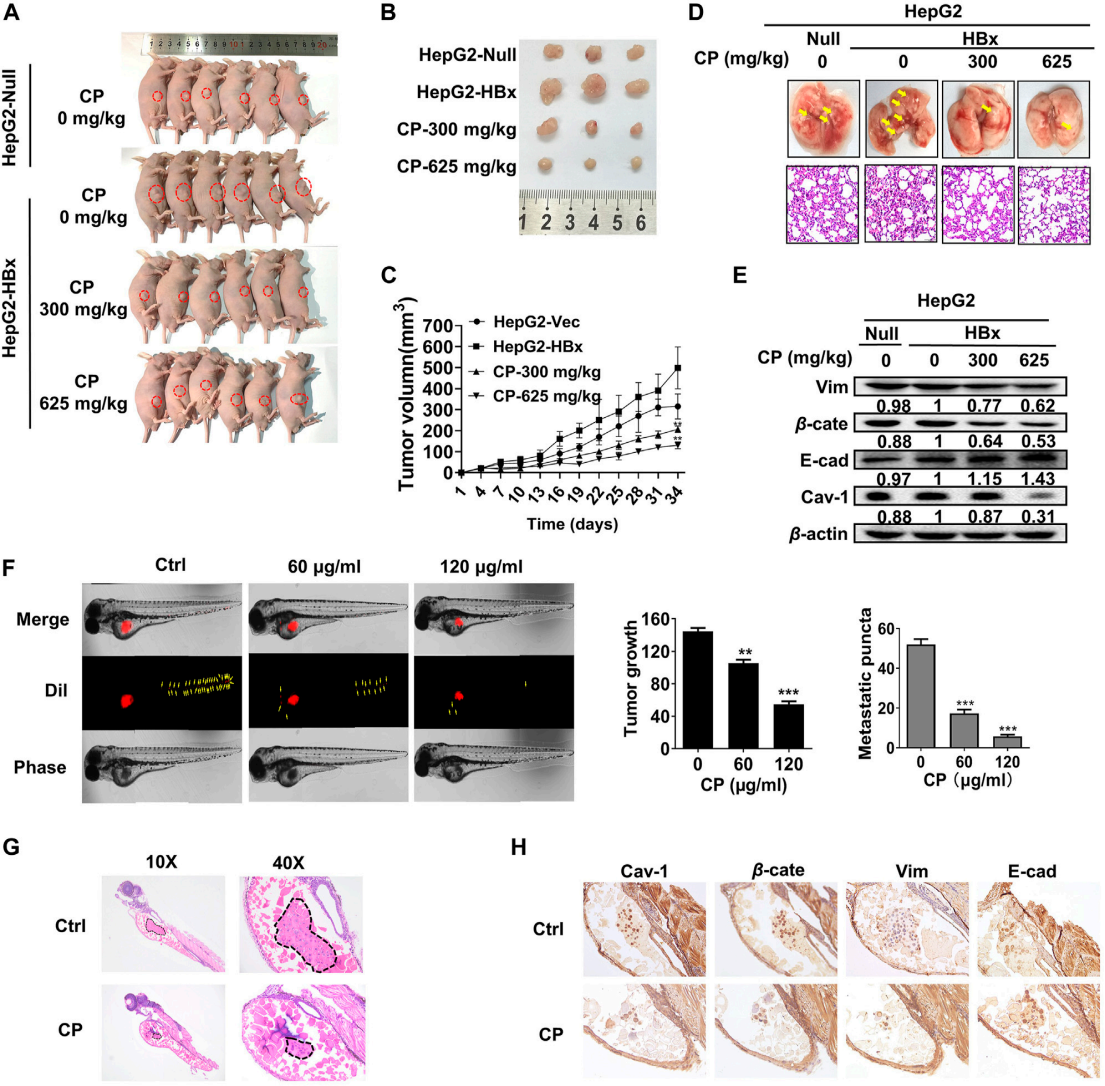
CP inhibited proliferation and migration of HBV-associated HCC *ex vivo* and *in vivo*. **(A–C)** HepG2-null and HepG2-HBx cells were grafted to the flank of nude mice by injecting the cells subcutaneously to observe tumor development, and volumes of tumors were determined at 3-day intervals. **(D)** Mice model with lung metastasis was established by tail vein injection of cancer cells. Upper panel, metastatic nodules in lungs. Lower panel, H&E staining of lung tissues. **(E)** Western blot analysis of tumor tissue showed that CP treatment decreased the expression levels of β-catenin and Cav-1, and enhanced the EMT process. **(F)** CP at concentrations from 60 to 120 μg/ml significantly decreased the primary as well as metastatic lesions in the zebrafish body when compared with control group. **(G)** H&E staining showed that CP suppressed the tumor growth in zebrafish. **(H)** Immunohistochemical staining of tumor in zebrafish showed CP treatment dramatically downregulated the expression levels of β-catenin, Cav-1 and Vimentin, and upregulated the expression level of E-cadherin. All values represented the means ± SD (*n* = 6, *p < 0.05, **p < 0.01, ***p < 0.001).

We next investigated the anti-growth and anti-metastasis potential of CP in nude mice. First, HepG2-HBx cells were subcutaneously injected into nude mice for the primary xenograft establishment with HBV-associated HCC. As shown, CP reduced tumor sizes (Figures 8A,B) and suppressed tumor growth in a dose dependent manner (Figure 8C), which was consistent with the *in vitro* experiments. There was a significant reduction in average tumor volume of CP-treated groups (130.45 ± 16.50 mm^3^ for 625 mg/kg CP high-dosage group, 206.54 ± 15.25 mm^3^ for 300 mg/kg CP low-dosage group) compared with that of the untreated group (498.95 ± 100.06 mm^3^) of mice bearing HepG2-HBx cells. To further explore the anti-metastasis effect of CP *in vivo*, a mouse model with pulmonary metastasis was established by tail vein injection of cancer cells. As shown in Figure 8D, fewer metastatic nodules in the lungs were observed in the CP administration group than in the control group. H&E staining of lung tissue showed that untreated group exhibited the morphological characteristics of cancerous tissues, whereas the CP treatment group did not lead to abnormal alterations of lung tissues. Tumor tissues were also collected for detecting expression levels of β-catenin, Cav-1, as well as metastatic markers by Western blotting analysis, and it was found that CP administration led to a reduction in the expression levels of β-catenin, Cav-1 and Vimentin as well as an increase in E-cadherin level (Figure 8E).

To further investigate the anti-metastatic effects of CP, we employed a novel *ex vivo* model of metastatic zebrafish xenotransplantation model. As shown in Figure 8F, untreated tumor cells rapidly spread away from the original microinjection position in zebrafish of the control group; In contrast, CP administration not only suppressed primary tumor marked by Dil staining, but also tended to reduce the spreading inclination toward the whole zebrafish body. H&E staining also showed that CP suppressed the tumor growth in zebrafish (Figure 8G). Pathological detection of tumor tissues confirmed that the anti-cancer activities of CP were tightly associated with downregulated expressions of β-catenin, Cav-1, and Vimentin, and upregulation in the expression of E-cadherin (Figure 8H). Collectively, these results suggested that CP played a role in inhibiting growth and metastasis of HBV-associated HCC *in vivo* and *ex vivo*.

## Discussion

TCM-derived herbs are promising medications for preventing and treating HBV-associated HCC due to their priority of multi-substance and multi-target features, accessible bioavailability and safety performances for preventing and treating HBV-associated HCC. In our previous experimental and clinical studies, TCM formula CP potently suppressed HBV-associated HCC by interfering with HBx-SHH pathway axis, inhibiting HBV-DNA replication and suppressing HBV-associated oncogenes (Tong et al., 2014; Li et al., 2019). However, there is still limited information regarding the pharmacologic action and underlying mechanisms of CP particularly during HBV-associated HCC metastasis. In this study, we demonstrated that CP efficiently inhibited growth and metastasis of HBV-associated HCC both *in vitro* and *in vivo.* To unravel the anti-metastasis mechanism of CP, we performed network pharmacology analysis of CP. The results revealed that a total of 76 major ingredients in CP formula. We also performed HPLC analysis for quality control and determination of the amount of the selected ingredients. The Venn diagram of the intersection among active components searching of literature review, network pharmacology analysis and HPLC analysis showed that there were several common active components including gallic acid (GA), luteolin, calycosin-7-glucoside and so on. Upon reviewing the literature, we noticed that GA possessed anti-HCC ability and showed regulatory capability toward β-catenin, which might play important roles in the efficacy of CP. Retrospectively, *Hadeer A Aglan et al.* emphasized that GA administration abrogated the hepatocarcinogenic effect of N-nitrosodiethylamine *in vivo* (Aglan et al., 2017). Compared with HL-7702 normal human hepatocytes, GA exerted better selective toxicity for HCC cells and inhibited the proliferation and apoptosis of HCC cell SMMC-7721 (Sun et al., 2016). Moreover, GA could activate the melanogenesis signaling cascade *via* inhibition of Wnt/β-catenin signaling pathways (Su et al., 2013). Therefore, GA has considerable potential to suppress HCC. The underlying mechanism might correlate with the regulation of β-catenin. For deeper research in the years following, we will continue to examine the role of GA in tumorigenesis and development of HBV-related HCC.

To explore the mechanism, we employed network-based pharmacology by searching the available public databases and systematic analysis. We identified 181 potential genes strongly associating CP with HBV-associated HCC. Among them, CAV-1 was regarded as one of core functional cancer-associated genes of HBV-associated HCC. KEGG enrichment analysis further suggested that the Wnt/β-catenin signaling pathway was implicated in the anti-cancer and anti-metastasis mechanisms of CP during progression of HBV-associated HCC (Miyazawa et al., 2004; Shackel, 2007; Zeng et al., 2007; Perugorria et al., 2019). We next demonstrated that Cav-1 suppression by CP probably resulted in β-catenin destabilization, subsequently exerting an anti-metastatic effect on HBV-associated HCC. This finding suggested that network-based pharmacology combined with bioinformatics analysis was reliable and provided a good complement to our research work. Retrospectively, a series of TCM studies using this methodology in liver cancer has been reported extensively. For instance, Jihan Huang et al. demonstrated the underlying mechanisms of Huachansu Capsules (HCSCs) on HCC *via* network pharmacology and pharmacological evaluation, and found 82 related anti-HCC targets and 14 potential pathways from HCSCs, suggesting the anti-HCC effects of HCSCs exhibited a multi-component, multi-target, and multi-pathway manner (Huang et al., 2020). By establishing the target-pathway network, Wei Guo et al. identified 42 bioactive chemicals as major anti-cancer components of TCM formula Zuojin Pill by interfering with multiple pathways, such as EGFR/MAPK, PI3K/NF-κB, and CCND1, in the prevention and treatment of HCC (Guo et al., 2019). Apart from the core target Cav-1 and the key pathway Wnt/β-catenin signaling, our computational analysis also suggested that the ant-metastasis activities of CP were partly due to the interactions of other molecule targets (e.g., Myc, TP53, and EGFR) and pathways (e.g., p53 signaling, PI3K-Akt signaling, and MAPK signaling), which needs to be further investigated and validated. Collectively, an integrated approach combining the bioinformatic analysis with pharmacological experiments may provide a robust method for molecular mechanism research of the effect of TCM formula on HCC (Wang et al., 2020).

In addition to network-based pharmacology, we also established zebrafish xenograft model for better understanding the anti-metastatic ability of CP. Zebrafish have recently become a hot animal model in tumor research with many attractive attributes (Goessling et al., 2007; Stoletov and Klemke, 2008). The genome sequence of the zebrafish is well-documented (http://www.ncbi.nlm.nih.gov/genome/guide/zebrafish) and shares 75% homology with human genome (Chakraborty et al., 2016). Genes in human malignancy are structurally and functionally conserved in zebrafish (Kim et al., 2017). Compared with mice, zebrafish is characterized by small size, short breed cycle, big spawn amount and transparent embryos, requiring minimal care and low cost of human and material resources. Zebrafish is also considered as a stable and comparable model to evaluate the physiological response to various pharmacologically active substances (Zon and Peterson, 2005). Previous researchers have developed a series of tumor transplantation assays in zebrafish models to investigate different pathological activities including cell migration, proliferation, angiogenesis and tumor cell extravasation (Lawson and Weinstein, 2002; Mathias et al., 2007; Stoletov and Klemke, 2008; Chen and Zon, 2009; Ellett and Lieschke, 2010). Pradip Shahi Thakuri et al. injected labeled breast cancer cells into zebrafish embryos to establish *in vivo* tumor metastasis model and found that fisetin significantly inhibited the metastasis of cells to the tail, which further confirmed that fisetin effectively suppressed the migration of metastatic triple negative breast cancer cells *in vivo* (Shahi Thakuri et al., 2020). Yvette Drabsch et al. injected cancer cells into the embryonic circulation (duct of Cuvier), then examined their metastasis into the avascular collagenous tail after administration of various substance to block the TGF-β signaling pathway. Their results showed that TGF-β receptor kinase inhibitors and tumor specific Smad4 knockdown suppressed the invasion and metastasis of breast cancer in zebrafish xenograft model (Drabsch et al., 2013). In this study, we established zebrafish xenotransplantation model through transplanting HCC cells into 2-day-old zebrafish embryos. We found that CP treatment significantly reduced the number of cells migrated to the tail, confirming the anti-metastasis function of CP formula *ex vivo*. We plan to additionally evaluate the safety issue of CP with the zebrafish-embryonic model in the future.

Furthermore, it was found that CAV-1 might be the hub gene contributing to the anti-metastasis effects of CP against HBV-associated HCC. In fact, Cav-1, the primary protein component of caveolae, has been reported to be a significant regulator in tumorigenesis and metastasis (Wang et al., 2020). Cav-1 is involved in the oncogenesis and development of various malignancies, such as colon, ovarian, lung and breast carcinoma (Joo et al., 2004; Li et al., 2009; Fecchi et al., 2012; Nimri et al., 2013). In this study, Cav-1 overexpression promoted metastasis potential of HBV-associated HCC by stabilizing β-catenin, while CP administration induced autophagic degradation of Cav-1, activated the Akt/GSK3β-mediated proteasome degradation of β-catenin *via* ubiquitination activation, and subsequently attenuated the metastasis-promoting effect of Cav-1. On one hand, our study revealed that the upstream mediation of CP on Cav-1 expression was autophagy-dependent. In fact, autophagic degradation of Cav-1 was reported to contribute to various biological processes. For example, the autophagic degradation of Cav-1 was activated by palmitic acid and led to apoptotic cell death and inflammation in hippocampal astrocytes (Chen et al., 2018). It also triggered the defenestration of liver sinusoidal endothelial cells through suppressing the NO-dependent pathway and F-actin remodeling (Luo et al., 2018). In our study, CP decreased the expression of Cav-1, which was predominantly associated with its autophagic activation. The additional evidence came from that increased co-expressions of Lamp1 and Cav-1 in CP-treated group, probably suggesting that the CP administration led to a translocation and accumulation of Cav-1 to lysosomes for autophagic degradation. On the other hand, we found that the downstream effect of CP on Cav-1 expression is β-catenin-dependent, subsequently endowing CP with the anti-metastasis effects in HBV-associated HCC. Thus, we next investigated the interaction between Cav-1 and β-catenin with or without CP treatment in HBV-associated HCC. We found that the high Cav-1 expression might favored the stabilization of β-catenin, and promoted β-catenin nuclear translocation for transcription of its downstream genes related to metastasis. In contrast, CP administration decreased Cav-1 to subsequently promoted the ubiquitination-activated proteasome degradation of β-catenin, resulting in the suppression of cancer metastasis. Previously, there have been similar studies on the associations between Cav-1 and β-catenin during cancer initiation and progression. Cav-1 activated the Wnt/β-catenin pathway by promoting Met expression, thereby facilitating cisplatin resistance of gastric cancer cells (Wang et al., 2020). In addition, Cav-1 also targeted WNT6, an activator of Wnt pathway, to induce chemoresistance to epirubicin in human gastric cancer cells (Yuan et al., 2013). It has also been reported that Cav-1 induced the EMT process in pancreatic cancer cells by increasing the expressions of plasma membrane bound E-cadherin and β-catenin, leading to tumor metastasis and chemoresistance (Salem et al., 2011). In HCC with HBx truncated at C-terminus (HBxΔC), Cav-1 was upregulated at the transcriptional level, thereby promoting cancer aggressiveness *via* the LRP6/β-catenin/FRMD5 signaling axis (Mao et al., 2019). Our study suggested that CP administration induced autophagic degradation of Cav-1, subsequently activated the Akt/GSK3β-mediated proteasome degradation of β-catenin *via* ubiquitination activation, and resulted in suppressing metastasis-promoting effect of Cav-1 in HBV-associated HCC.

## Conclusion

In conclusion, the current study demonstrates that autophagic inhibition of Cav-1 by CP activates ubiquitination and proteasome degradation of β-catenin to suppress metastasis in HBV-associated HCC. These findings provide evidence-based support for the clinical application of CP.

## Data Availability

The original contributions presented in the study are included in the article/Supplementary Material, further inquiries can be directed to the corresponding authors.
