# Supplementary figures and images for: Autophagic Inhibition of Caveolin-1 by Compound Phyllanthus urinaria L. Activates Ubiquitination and Proteasome Degradation of β-catenin to Suppress Metastasis of Hepatitis B-Associated Hepatocellular Carcinoma
Source: Front Pharmacol. 2021 Jun 8;12:659325. doi: 10.3389/fphar.2021.659325 (PMC8217966; doi:10.3389/fphar.2021.659325)

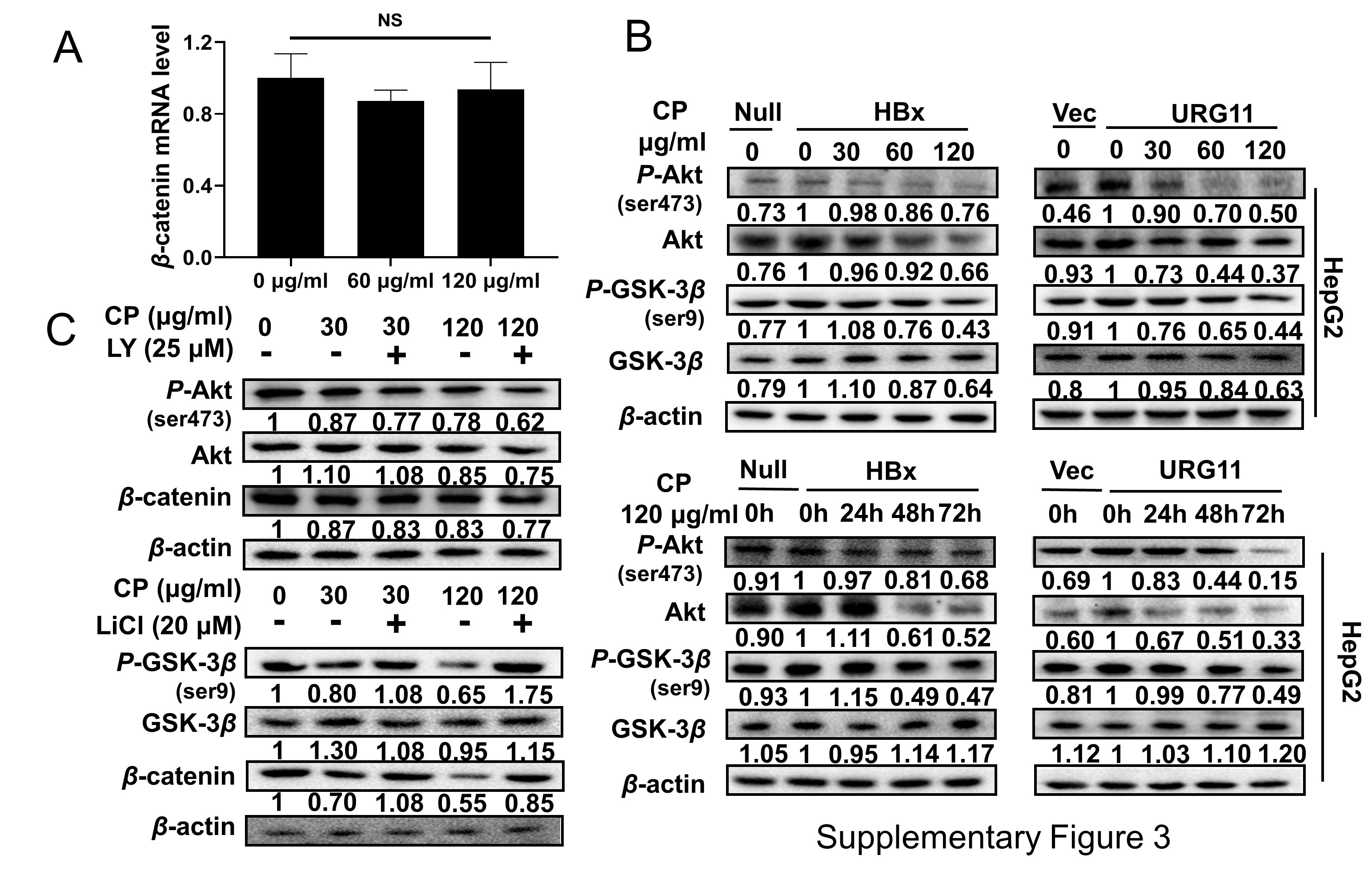

Supplement: Supplementary file 1 [file Image3.JPEG]

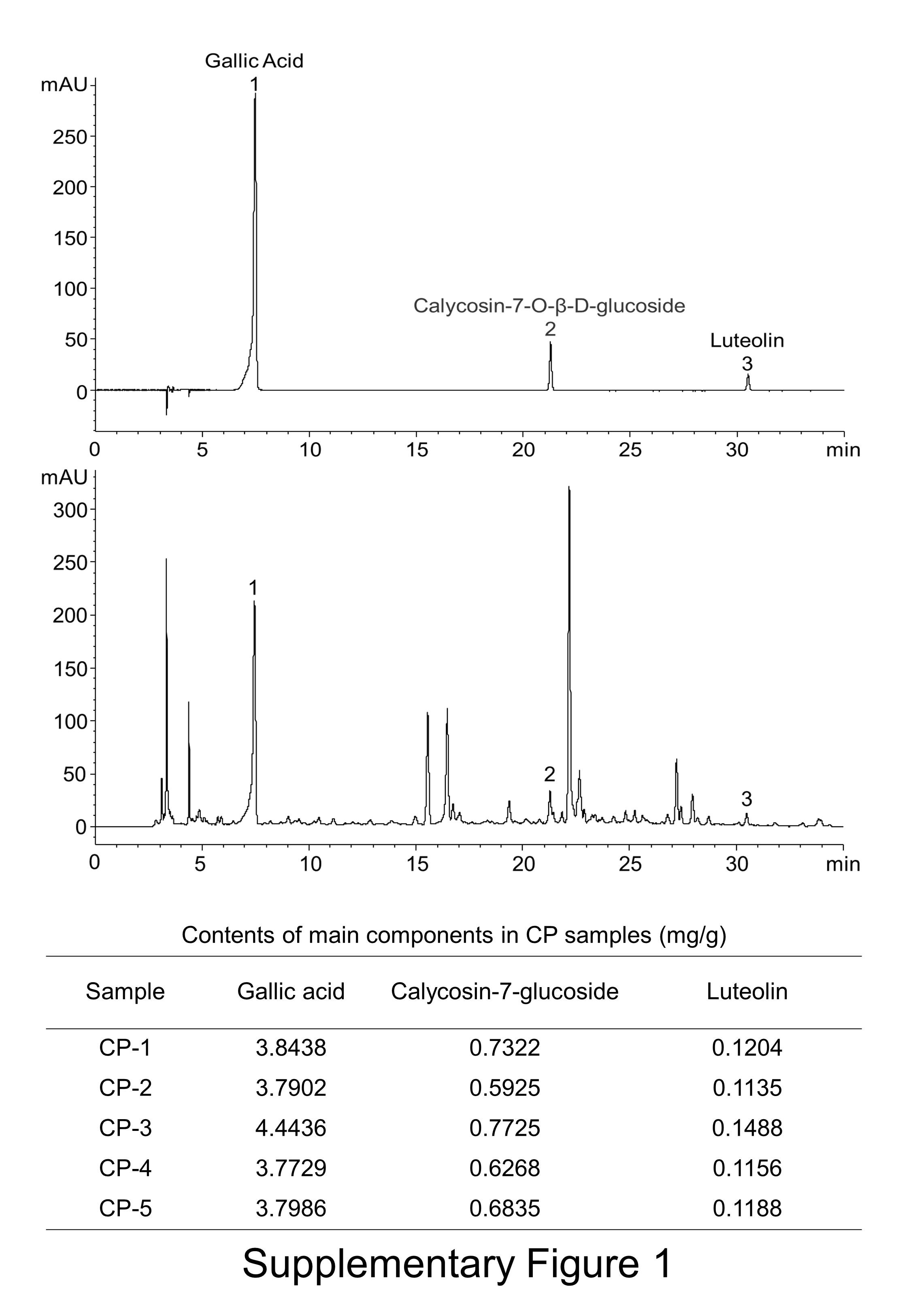

Supplement: Supplementary file 3 [file Image1.JPEG]

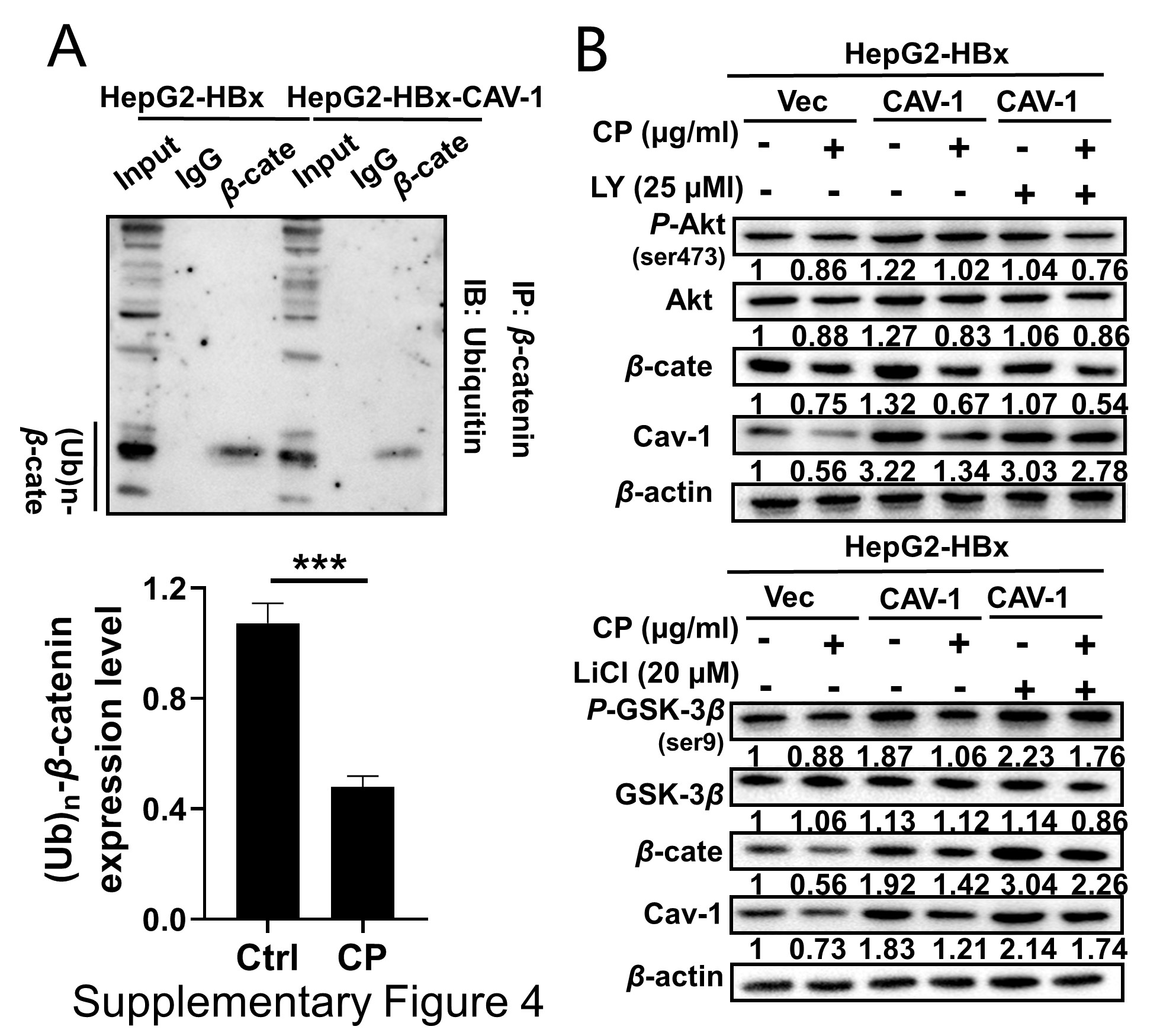

Supplement: Supplementary file 4 [file Image4.JPEG]

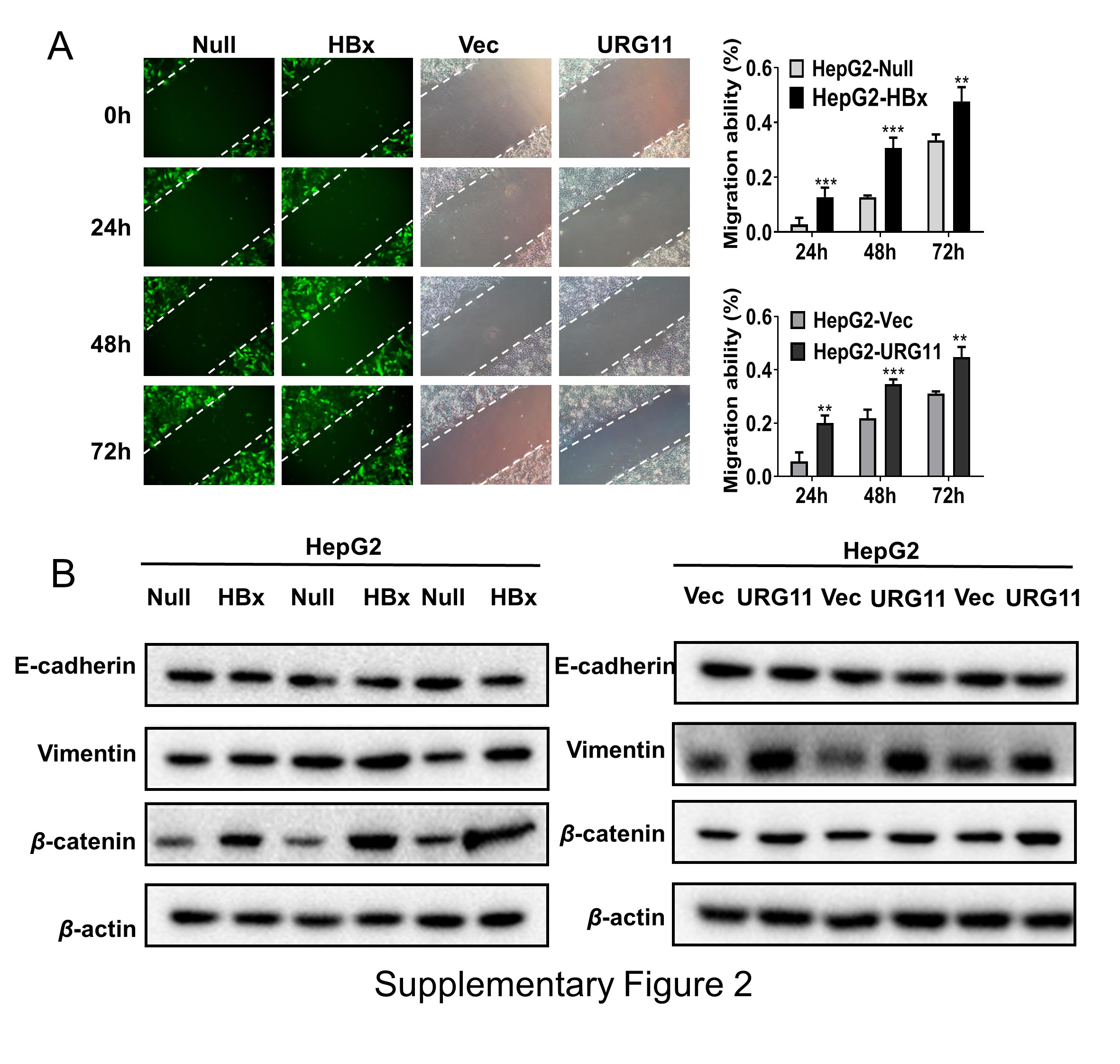

Supplement: Supplementary file 5 [file Image2.JPEG]
