# Supplementary material for: Autophagic Inhibition of Caveolin-1 by Compound Phyllanthus urinaria L. Activates Ubiquitination and Proteasome Degradation of β-catenin to Suppress Metastasis of Hepatitis B-Associated Hepatocellular Carcinoma
Source: Front Pharmacol. 2021 Jun 8;12:659325. doi: 10.3389/fphar.2021.659325 (PMC8217966; doi:10.3389/fphar.2021.659325)
Supplement: Supplementary file 2 [file Table1.docx]

**Supplementary Table 1. Selectivity Index of CP on three HCC cell lines**.

|  | HL-7702 | HepG2 | SMMC-7721 | Huh-7 |
| --- | --- | --- | --- | --- |
| IC_50_ (μg/ml) | 1495.57 | 159.00 | 180.58 | 201.25 |
| SI^*^ |  | 9.41 | 8.28 | 7.43 |

^*^SI: Selectivity Index. SI=IC50^HL7702^/IC50^HCC cell line^
